# Connected Speech in Neurodegenerative Language Disorders: A Review

**DOI:** 10.3389/fpsyg.2017.00269

**Published:** 2017-03-06

**Authors:** Veronica Boschi, Eleonora Catricalà, Monica Consonni, Cristiano Chesi, Andrea Moro, Stefano F. Cappa

**Affiliations:** ^1^NETS, Center for Neurocognition, Epistemology and Theoretical Syntax, Institute for Advanced Study-PaviaPavia, Italy; ^2^Third Neurology Unit and Motor Neuron Diseases Center, IRCCS Foundation “Carlo Besta” Neurological InstituteMilan, Italy; ^3^IRCCS S. Giovanni di Dio FatebenefratelliBrescia, Italy

**Keywords:** connected speech, linguistic domains, linguistic variables, neurodegenerative disorders, review, picture description, story narration, interview

## Abstract

Language assessment has a crucial role in the clinical diagnosis of several neurodegenerative diseases. The analysis of extended speech production is a precious source of information encompassing the phonetic, phonological, lexico-semantic, morpho-syntactic, and pragmatic levels of language organization. The knowledge about the distinctive linguistic variables identifying language deficits associated to different neurodegenerative diseases has progressively improved in the last years. However, the heterogeneity of such variables and of the way they are measured and classified limits any generalization and makes the comparison among studies difficult. Here we present an exhaustive review of the studies focusing on the linguistic variables derived from the analysis of connected speech samples, with the aim of characterizing the language disorders of the most prevalent neurodegenerative diseases, including primary progressive aphasia, Alzheimer's disease, movement disorders, and amyotrophic lateral sclerosis. A total of 61 studies have been included, considering only those reporting group analysis and comparisons with a group of healthy persons. This review first analyzes the differences in the tasks used to elicit connected speech, namely picture description, story narration, and interview, considering the possible different contributions to the assessment of different linguistic domains. This is followed by an analysis of the terminologies and of the methods of measurements of the variables, indicating the need for harmonization and standardization. The final section reviews the linguistic domains affected by each different neurodegenerative disease, indicating the variables most consistently impaired at each level and suggesting the key variables helping in the differential diagnosis among diseases. While a large amount of valuable information is already available, the review highlights the need of further work, including the development of automated methods, to take advantage of the richness of connected speech analysis for both research and clinical purposes.

## Introduction

The detection and characterization of language impairments play an increasingly important role in the identification and diagnosis of many neurodegenerative diseases. Language deficits are present in several neurodegenerative pathologies, sometimes in the early stages, as a selective and prominent symptom, such as Primary Progressive Aphasia (PPA), or in combination with other cognitive disorders, such as, in Alzheimer's disease (AD).

A progressive, selective language disorder is the core feature of PPA: a set of syndromes due to different neurodegenerative diseases (for reviews, Mesulam et al., 2014; Cerami and Cappa, 2016), often related to fronto-temporal lobar degeneration (FTD). Current diagnostic criteria distinguish three different variants: the non-fluent, the semantic, and the logopenic variant, each of which tends to exhibit specific patterns of linguistic deficits and a characteristic distribution of brain atrophy (Gorno-Tempini et al., 2011). People suffering from the non-fluent variant are characterized by effortful speech, presenting morpho-syntactical deficits and omission of function words leading to agrammatism and oversimplification of language output and/or apraxia of speech, resulting in loss of prosody and articulatory errors. Patients with the semantic variant show severe anomia and consistent difficulties in comprehending single words, deficits attributed to the degradation of semantic representations. Person suffering from the logopentic variant present marked word-finding difficulties, difficulties in sentence repetition, in the absence of agrammatism, apraxia of speech, and semantic memory impairment.

Patients with “typical” AD may also show language impairment in the early stages of the disease, although the prominent impairment concerns episodic memory and the progression of the disease leads to deficits in several other cognitive domains. Language deficit primarily occurs because of a decline in lexical semantic abilities, with anomias and semantic paraphasias, word comprehension, and verbal fluency impairment (Forbes-McKay and Venneri, 2005; Taler and Phillips, 2008; Catricalà et al., 2014, 2015a). Disorders at pragmatic level of language processing, namely alteration in discourse planning, have also been reported (Chapman et al., 1998, 2002). Phonological and syntactic processing is relatively spared, at least in early stages (Kavé and Levy, 2003; Forbes-McKay and Venneri, 2005; Tang-Wai and Graham, 2008), although some studies reported a simplification of syntax (Kemper et al., 1993; Ripich et al., 2000; Altmann et al., 2001) and impairment in phonological structure (Croot et al., 2000). With the progression of the disease, a severe language impairment becomes pervasive in AD, with speech restricted to echolalia and verbal stereotypes (Ferris and Farlow, 2013). Patients with amnestic Mild Cognitive Impairment (aMCI; Petersen, 2004), considered to be often a prodromal stage of AD, show language impairments very similar to those described in the early stages of AD, involving lexico-semantic and pragmatic domains (Duong et al., 2006; Taler and Phillips, 2008; Tsantali et al., 2013; Drummond et al., 2015).

Parkinson's Disease (PD) is often associated with a disruption of the motor speech, affecting respiration, phonation, articulation, resonance and prosody (Goberman and Coelho, 2002). Language deficits involve morpho-syntactic processing, in particular verb inflection (Ullman et al., 1997), verb generation (Péran et al., 2003; Crescentini et al., 2008), and sentence comprehension (Grossman, 1999). Pragmatic difficulties affect the abilities to understand metaphoric meanings, inferences (Monetta and Pell, 2007; Assal and Ghika, 2013), and ironic remarks (Monetta et al., 2009).

Language impairment does not constitute a central feature for Dementia with Lewy bodies (LBD, McKeith et al., 2005). Naming and verbal fluency deficits have however been reported and attributed to executive deficits (Galasko et al., 1996; Delbeuck et al., 2013). The speech output of LBD patients is abnormally slow, with speech sound errors that may be due in part to the motor disorder (Ash et al., 2011, 2012a). Syntactic and pragmatic difficulties have been described, with reduced syntactic complexity in production and grammatical comprehension and difficulty in organizing narrative speech (Ash et al., 2011, 2012a,b; Grossman et al., 2012). About one third of patients with Cortico-basal Syndrome (CBS) shows language deficits, and their incidence may increase as the syndrome progresses (McMonagle et al., 2006). Language dysfunction in persons with CBS has been described either in the form of progressive non-fluent aphasic phenotype (Burrell et al., 2014) or as a milder language dysfunction involving a higher proportion of patients (Graham et al., 2003; McMonagle et al., 2006). This subtle language impairment involves mainly phonological processing (Graham et al., 2003), and syntactic knowledge (Cotelli et al., 2007), whereas semantics is normal or only mildly impaired (Graham et al., 2003).

Speech and language impairments have been described in patients with Huntington's disease (HD), as a central feature of the development of dementia syndrome (Ludlow et al., 1987; Podoll et al., 1988), due to the spread of degeneration within the striatum and toward cortical regions. In the early stages, patients with HD might present with a loss of conversational initiative in combination with a simplified syntactical structure of spontaneous speech (Podoll et al., 1988; Illes, 1989). Subtle deficits in word morphology and sentence comprehension have been linked to impairment of language rules application due to striatal involvement (Teichmann et al., 2008; see however Longworth et al., 2005). Mild dysfunctions in lexical operations, such as word retrieval and inhibition of inappropriate items, have been found in people suffering from HD (Longworth et al., 2005) and linked to failures in inhibiting competing alternatives. Naming disorders have been also documented and attributed to visuo-perceptive impairments (Podoll et al., 1988; Hodges et al., 1991), whereas the structure of semantic memory appears to be essentially intact (Podoll et al., 1988).

While limited attention has been paid to the language domain in Amyotrophic Lateral Sclerosis (ALS), some studies have identified language deficits in non-demented ALS patients suggesting the existence of multiple cognitive phenotypes (Consonni et al., 2016). Individuals with ALS may present articulation difficulties and syntactic processing deficits in the forms of simplified syntax (Tsermentseli et al., 2016) and syntactic comprehension (Yoshizawa et al., 2014). Semantic and pragmatic impairments are also common. The first is characterized by word finding difficulty, deficits in verbal fluency, naming and single word comprehension (Phukan et al., 2007; Taylor et al., 2013; Leslie et al., 2015). Pragmatic impairment is attested by the failure of ALS patients to maintain the discourse topic, to provide the appropriate amount of information and salient elements, and to recall non-explicit information and non-literal meanings (Ash et al., 2014; Bambini et al., 2016).

From the brief overview provided above, it is clear that an analysis of language performance can be considered as an integral part of the cognitive evaluation of patients affected by a wide array of neurodegenerative conditions. It is thus surprising that the standard cognitive assessment of these patients often includes only measures of single word processing, such as picture naming or verbal fluency. The most informative part of language assessment, i.e., the analysis of extended speech production, is often neglected, or performed according to non-standardized procedures. The analysis of connected speech is a very useful tool, providing detailed data about all linguistic levels: phonetic, phonological, lexico-semantic, morpho-syntactic, syntactic and discourse-pragmatic. Connected speech has been used to successfully distinguish patients with different neurodegenerative diseases from non-brain-damaged persons (Gross et al., 2010; Ash and Grossman, 2015; Drummond et al., 2015; Fraser et al., 2015a; Tsermentseli et al., 2016) and to identify language characteristics associated with specific neurodegenerative diseases (Wilson et al., 2010; Ash et al., 2011, 2012a, 2013). The literature focused on connected speech analysis has proposed a large number of linguistic variables to analyze speech production, but the heterogeneity of the variables adopted and the way they are measured and classified hinder the generalization and comparison of data.

This review aims to show the state of the art of the evaluation of language through connected speech analysis in the most prevalent neurodegenerative diseases. First it analyzes the differences in the tasks used to elicit connected speech, namely picture description, story narration, and interview, discussing the possible different specificities in assessing distinct linguistic domains. Then we examine the linguistic variables used to assess connected speech in the above mentioned diseases. We first defined the linguistic variables adopted in all studies, in an effort to overcome the heterogeneity of nomenclatures and measurements. In addition, we propose a general descriptive classification encompassing five different linguistic levels to promote uniformity in data collection and analysis (Table 1). Finally, we highlight the linguistic domains most affected by each different neurodegenerative disease, indicating the variables consistently impaired at each level, aiming at identifying those that appear to be most suitable to distinguish each pathological condition from healthy persons and for the differential diagnosis among different pathologies.

**Table 1:** **Tasks, pathologies and linguistic levels of the variables considered in the review**.

**Classification parameter**	**Description**
Pathology	Primary Progressive Aphasia (PPA)
	Alzheimer's Disease (AD)
	Mild Cognitive Impairment (MCI)
	Parkinson's Disease (PD)
	Parkinson's Disease with Dementia (PDD)
	Corticobasal syndrome (CBS)
	Dementia with Lewy Body (LBD)
	Huntington's Disease (HD)
	Amyotrophic Lateral Sclerosis (ALS)
Task	Picture Description
	Story Narration
	Interview
Linguistic levels	Phonetic-phonological
	Lexico-semantic
	Morpho-syntactic
	Syntactic
	Discourse-pragmatic

### Literature review process

#### Methods

An exhaustive search has been conducted using electronic online databases: PubMed, SCOPUS, World Wide Sciences, Google Scholar, using different combinations of the following search terms: connected speech, spontaneous speech, speech production, narrative speech, picture description, interview, semi-structured interview, linguistic features, language impairment, dementia, neurodegenerative disease, PPA, semantic dementia, logopenic variant, progressive non-fluent aphasia, Mild Cognitive Impairment, Alzheimer's Disease, Lewy Body Dementia, ALS, Parkinson's Disease, PD with dementia, Parkinsonism, Corticobasal Syndrome, Progressive Supranuclear Palsy, automated analysis.

The bibliographic search yielded a total of 106 papers. We considered studies including the following pathological conditions: amnestic Mild Cognitive Impairment, Alzheimer's Disease, PPA, movement disorders, and ALS (see Table 1). We considered only studies based on a group study, including at least six subjects and a comparison with a group of healthy persons. No results on single cases have been included. Only studies focusing on connected speech through a picture description, a story narration, or an interview have been considered, excluding narrative recall task as it implies memory processes that could influence speech production, creating a confound, especially in diseases with a memory deficit. No other tasks encouraging speech production as naming, fluency or reading were considered. We included only studies reporting detailed results for the main linguistic variables and we excluded review articles. The resulting 61 articles were considered for the present review.

## Task used to elicit speech samples

Different tasks have been used to elicit connected speech in neurodegenerative diseases. In most cases, they were originally developed for the assessment of extended production in vascular aphasia. The speech elicited using situational pictures and story narration tasks can be classified as semi-spontaneous speech, as these tasks impose a sort of structure to the speech output (Prins and Bastiaanse, 2004), i.e., restricting the description to specific information conveyed by the pictures or the predefined stories. Interviews and conversations are generally considered spontaneous speech productions, as the subjects are less restricted in their answer, both in terms of quantity of information and time.

In the picture description task, participants are supplied with a picture, depicting simple or complex scenes, which they are asked to describe. This task requires minimal instructions and interventions by the examiner. The duration of the task is ~2–5 min in healthy subjects. This assignment imposes a predictable speech output, as the description should contain key elements, information, or semantic units (subjects, objects, actions, places), represented in the picture. Consequently, the speech obtained can be scored by measuring the number of correct information units identified. The use of picture description appears to facilitate the assessment of the lexico-semantic level, especially encouraging the usage of nouns and deixis (March et al., 2006). Accordingly, it is usually employed to identify semantic deficits and word retrieval difficulties (Sajjadi et al., 2012a). Conversely, the variety of syntactic structures elicited is usually limited, and mainly restricted to simple constructions: declarative present tense statements are generally sufficient to describe the picture (i.e., “The woman is washing dishes; the boy is falling off the stool,” example taken from Garrard and Forsyth, 2010). The most used picture description tasks employed in the assessment of extended speech in neurodegenerative diseases are the Cookie Theft of the Boston Diagnostic Aphasia Examination (Goodglass et al., 1983), and the Picnic scene of Western Aphasia Battery (WAB) (Kertesz, 1982), see Table 2 for the complete list.

**Table 2:** **Schematic description of the materials, different versions, advantages and limitations of each task employed for the assessment of connected speech in neurodegenerative diseases**.

**Task**	**Material**	**Test**	**Advantages**	**Limitations**
**SEMI-SPONTANEOUS SPEECH**				
Picture description	Simple and complex pictures	*The Cookie Theft* (Boston Diagnostic Aphasia Examination, Goodglass et al., 1983) *The Tripping Woman Picture* (Semenza and Cipolotti, 1989) *The Traffic Chaos Picture* (Forbes-McKay and Venneri, 2005) *The Bus Stop Picture* (Forbes-McKay and Venneri, 2005) *Picnic scene* (Western Aphasia Battery, Kertesz, 1982) Picture from Comprehensive Aphasia Test (Swinburn et al., 2004) *Bank robbery* (Nespoulous et al., 1992)	Easy procedure of administration Short task duration Useful to assess lexico-semantic deficits Easy scoring of the predefined contents of the picture Easy comparison across subjects and languages	Limited variety of syntactic structures Reduced discourse and narrative information
Story narration	Wordless picture book pictorial cards	*Cinderella* story Children's book: *Frog, Where Are You?*, (Mayer, 1969) *The Dog Story* (Le Boeuf, 1976) *Car accident* (Ska and Duong, 2005) *Peanuts* (Schulz, 1976)	Easy scoring of the predefined contents and event sequences Useful to assess lexico-semantic deficits Useful to assess complex syntactic structures Useful to assess discourse and narrative speech	Complex procedure of administration Long task duration Requires comprehension of the narrative stimuli
**SPONTANEOUS SPEECH**				
Interview	Semi-structured: predefined questions Unstructured: none	Autobiographical Memory Interview (AMI, Kopelman et al., 1990) Part 1 Western Aphasia Battery (WAB, Kertesz, 1982) *ad hoc* list of predefined open ended questions, topics: family, hobbies, career, etc.	Useful to assess discourse and pragmatic processing	Prone to inter-interviews differences Long task duration Difficult comparison across subjects and languages Very time-consuming output analysis

The story narration task is used to assess the participant's ability to elaborate a story characterized by a sequence of events or actions. It usually employs a wordless book or pictorial cards, where a sequence of pictures represents a story. Two main procedures are generally adopted. In the first procedure, participants are asked to look at the pictures of a wordless book and narrate the story starting from the first page/card and continuing through the book (Ash et al., 2014). The content of the story can vary, ranging from children's stories (i.e., children's book: “Frog, Where Are You?,” Mayer, 1969; comics: “Peanuts,” Schulz, 1976) to common events of daily life, as in the case of the “car accident” (Ska and Duong, 2005) or the “dog story” (Le Boeuf, 1976). In the second procedure, a wordless picture book, based on a famous story, i.e., Cinderella, is given to the participant; then the book is removed and the subject is asked to tell the story in his own words (Saffran et al., 1989; Fraser et al., 2014b). Both procedures require, in addition to producing the story components in a structured and coherent framework, the comprehension of the story characters and events, including temporal and spatial shifts as well as the goals and internal responses of the characters. The procedure in which visual stimuli are available to the subject reduces memory demands. In both conditions, the task duration is ~10–15 min. The fixed context defined by the event sequence represented in the picture book imposes a relatively structured speech output. It is possible to quantify the completeness of the narrative description/schema, since the events described should be told following a predefined temporal and sequential order, depending on the book's storyline (Duong et al., 2005). These characteristics allow to easily analyze discourse and pragmatic information (Drummond et al., 2015), lexical and semantic abilities, and syntactic complexity (de Lira et al., 2011).

The interview is used to elicit spontaneous speech production, employing questions to guide a conversation between speakers. There are three types of interviews: structured, semi-structured, and unstructured; the former, producing a very limited speech output, has never been used in neurodegenerative diseases for the assessment of spontaneous language. Semi-structured interviews include predefined open-ended questions, sometimes mixed to closed-ended questions. The purpose of a semi-structured interview is to discuss a topic in detail: sometimes the interviewer intervenes directing the subject into a specific topic area. Examples of standardized semi-structured interviews are the language assessment protocol of the WAB (Kertesz, 1982) and the Autobiographical Memory Interview (AMI; Kopelman et al., 1990). The latter was originally developed to test remote episodic and semantic personal memory. Unstructured interviews or informal conversations do not have a systematic format to follow; there is no predefined set of questions, but only wider themes to talk about. They usually involve familiar and generic topics concerning family, hobbies, career, etc. The interview usually starts with a very broad open ended question (i.e., Tell me about your family?) to introduce a topic and to encourage the conversation. Participants are free to answer as they like, thus reproducing a spontaneous conversation. Although the unstructured interview has been sometimes employed to assess language production in dementia (Singh et al., 2001; López-de-Ipiña et al., 2013), the semi-structured interview is preferred because of its semi-fixed structure that can be easily administrated and reproduced. In each case, however, responses are very different across participants and can be difficult to compare. The task duration is highly variable, ~5–20 min depending on the type of interview. Moreover, the analysis of speech output is very time-consuming and difficult to score in the absence of predefined task constraints. Both semi-structured and unstructured interviews may be useful in analyzing the discourse-pragmatic domain, highlighting cohesion, and coherence (Lai, 2014). They have been also used to identify alterations in syntactic and semantic processing (Ripich et al., 2000; Sajjadi et al., 2012a; Lai, 2014), even if anomia can be more easily compensated than in the case of the picture description tasks (Garrard and Forsyth, 2010).

To summarize, three classes of tasks, picture description, story narration, and interview, have been employed to assess connected speech in neurodegenerative diseases. They show several intrinsic differences, suggesting a different specificity for assessing distinct linguistic levels. A schematic description of the characteristics of each task is reported in Table 2.

Only a few studies targeting neurodegenerative diseases have directly compared these tasks. A direct comparison between a semi-structured interview and picture description in patients with the semantic, logopenic, and non-fluent variant of PPA, mixed PPA, and AD revealed that the semi-structured interview is more sensitive in highlighting alterations in discourse, and abnormalities of morphological and syntactical structure than the picture description. In contrast, picture description is more apt to assess lexico-semantic impairment than the semi-structured interview (Sajjadi et al., 2012a,b). Another study, comparing a picture description task (Cookie Theft, Goodglass et al., 1983) with a narrative description (Children's book: Frog, Where Are You?; Mayer, 1969), found that these tasks can be employed interchangeably for the assessment of phonetic, semantic, and syntactic domains in subjects with clinical diagnoses of non-fluent, logopenic, and semantic variant of PPA, behavioral variant of the frontotemporal dementia, and non-brain damaged persons (Ash et al., 2013).

## Definitions and classification of the linguistic variables

Linguistic variables, also known as features, are measures used to describe quantitative and qualitative aspects of language production. In this review we considered only the linguistic variables reported in the 61 studies and resulting as significant in at least one statistical comparison (pathological group vs. non-brain damaged group or between different pathological conditions). A total of 120 linguistic features have been included; a definition of each is reported in Supplementary Table 1. Five linguistic levels have then been used to classify the variables: phonetic-phonological, lexico-semantic, morpho-syntactic, syntactic, and discourse-pragmatic. This account describes only the level at which the variable is situated, respectively sound and/or phoneme, word, sentence and discourse, and does not indicate the nature of the deficit. In several cases, in fact, a pathological feature at a specific linguistic level may be caused by multiple different mechanisms of cognitive impairment, please see Supplementary Table 1 for examples.

Phonetic and phonological variables describe language production at the speech sound level. In this class we include, according to previous studies (Szatloczki et al., 2015), several acoustic measures, like the time employed to produce words, phonemes, syllables, or the quantification of pauses in the speech, the amount of time in the sample containing both speech and pauses (locution time), fillers, pauses, etc. These measures can certainly capture deficits affecting other linguistic levels. For example, these variables are also intrinsically related to discourse as pauses may reveal difficulties in discourse planning. Difficulties in fluency and discourse planning may be responsible for hesitations (fillers and pauses), which indicate some form of cognitive lapse, such as a failure to communicate properly. The meaning of fillers is related to their place in the discourse. When placed at the beginning or end of a dialog they could indicate a trouble in understanding something or the need to receive an input. They could also indicate the desire to take back what was said or to reword it. In these case fillers occur in the middle of a dialogue (Guinn and Habash, 2012). Pauses have a variety of interpretations and encompass several linguistic levels: difficulty in articulation, impairment in lexical access (word finding difficulty), deficits in syntax and discourse planning.

Another group of acoustic variables, only recently employed for the analysis of connected speech in pathological context, is Mel-frequency cepstral coefficients (MFCCs). These features have been used mainly for speech recognition, and measure the power spectrum of a speech signal: for example, the peakedness of the signal (kurtosis) or the lack of symmetry (skewness) (Fraser et al., 2014a). Speech production also offers information on intonational contour, syllabic stress, and duration. This prosodic information is measured on the basis of alterations in pitch, volume, and duration of the speech output. Speech sound errors include sounds that do not exist in the language of the speaker (phonetic errors), as well as substitutions, additions, and deletions of a well-articulated phoneme (phonemic errors). Another type of speech sound error is the false start, namely the production of partial words. This variable has been included among phonetic and phonological features, although it may also indicate an impairment at lexico-semantic level.

Lexico-semantic features capture impairments at word and content levels. Words can be classified using part-of-speech categories (i.e., open class and closed class words or more specifically noun, verb, etc.). The average rate of occurrence for each part-of-speech can be used to analyse the lexical distribution of words produced or to identify difficulties in accessing a specific word class (Thomas et al., 2005). Part-of-speech can be used to identify deixis, which represent a bridge between the communicative context and speakers (March et al., 2006), and include demonstratives (spatial deictics) and personal pronouns (person deictics). The production of deictic terms with no clear referents makes the discourse vague and ambiguous. Both deictic terms and open-class words have referential meaning in context, although deictic reference varies between contexts more than open-class word meaning (Altmann et al., 2001). Lexico-semantic variables may be used to capture lexical richness or to describe how informative is a discourse. Variables used to capture lexical richness of vocabulary are Type-token ratio, Brunét's index, and Honoré's statistic (Thomas et al., 2005; Guinn and Habash, 2012), each presenting different characteristics and specifications (see Supplementary Table 1 for details). Word frequency may indicate how informative is a discourse. For example, an overuse of content words with high frequency has been associated to less accurate speech (Fraser et al., 2015a). Errors at this level can be divided in lexical and semantic. The first include word-finding difficulties, indefinite terms, repetitions, revisions and neologisms (Croisile et al., 1996; de Lira et al., 2011). Sometimes neologisms are not distinguishable from complex phonemic paraphasias, because the intended target of a neologism may not always be apparent (Graham et al., 2004). Lexical errors may alter the clarity of speech production, influencing syntactic structure, and discourse planning. Semantic errors generally are substitutions of a word with another semantically related word, i.e., semantic paraphasias, and can involve superordinate (animal instead of cat) or coordinate terms (dog instead of cat).

Morpho-syntactic features usually report information on word inflection and agreement, such as tense, mood, aspect, person, number, and gender. Phonological processes are inherently connected to inflectional processes, as the allomorph selection depends on the phonological context. Inflection and agreement influence also the syntactic structure (Wilson et al., 2014, see also Moro, 2015). Morphological errors consist of the selection of an existent inappropriate morphological form of a word, as well as of the erroneous use of a non-existent word form. Grammatical errors involving morphology include the absence or inappropriate use of functors or an incorrect use of verbal tenses, which also reflects incorrect temporal cohesion at the discourse level.

At sentence level, linguistic variables, i.e., the number of words per clause, of utterances, of embeddings, of passive constructions, of dependent, and of simple clauses, provide a measure of the syntactic complexity of discourse. Syntactic errors include general structural violations and incomplete sentences (Kavé and Levy, 2003). These variables describe the type of syntax produced, but they may capture deficits at multiple linguistic levels. Incomplete sentences refer to sentences that are not correctly developed, since they are missing some fundamental part of the structure. Although this feature describes a syntactic phenomenon, it may be the result of impairment at different linguistic levels, reflecting deficits at lexico-semantic, syntactic, or discourse level.

Discourse and pragmatic features identify elements in the speech that contribute to the continuation of conversation and include cohesion, coherence, a correct use of pronouns, and conjunctions. These features measure how the context contributes to the meaning of the discourse produced and are used to calculate the appropriate amounts of information. Cohesion refers to the indicators of relations within and between sentences and may be distinguished in referential cohesion, temporal cohesion, and causal cohesion. Coherence may also be local or global. Local coherence indicates how close is the relation between an utterance and the preceding one. Global coherence indicates utterances closely associated with the general topic and measures disruptive topic shifts or digressions (Lai, 2014).

## Results: relevant features and linguistic profiles of the main clinical conditions

For each disease, the linguistic profile is described as a set of linguistic variables extracted from connected speech analyses. Only variables reported to be significant in distinguishing at least one specific disease from healthy persons or from other diseases were included. The features reported as significant in at least more than half of the studies (with at least three studies investigating the same feature) or in two out of two studies were considered as the most relevant in characterizing each profile. Table 3 reports the definitions of the 61 most relevant variables. Table 4 reports the results of the comparisons of each pathologic group with a group of non-brain damaged persons for the most relevant variables. Results for all the 120 variables are reported in the Supplementary Materials.

**Table 3:** **Summary of the 61 most relevant features and their definitions (see text for details)**.

**Linguistic level**	**Linguistic feature**	**Definition/how to measure**
**Phonetic and Phonological**	Speech rate	Number of words per minute
	Maximum speech rate	Average of the words per minute for the three most rapid sequences of 10 or more words
	Mean first autocorrelation function	The cross-correlation of a signal with itself at different points in time
	Number of pauses	Number of pauses produced
	Between–utterance pause duration	Proportion of total duration of pauses between utterances
	Hesitation ratio	Total duration of hesitations divided by the total speech time; an hesitation is defined as the absence of speech lasting more than 30 ms
	Phonemic errors	Well-articulated phoneme substitutions, additions, and deletions
	Standardized phonation time	Text length divided by the total phonation time
	Total locution time	The amount of time in the sample containing both speech and pauses
	F0 Standard Deviation (prosody)	Variations of fundamental frequency, vibration rate of vocal folds
	Intensity Standard Deviation (prosody)	Variations of average squared amplitude within a predefined time segment (“energy”) after removing any silence period exceeding 60 ms
	Filled pauses	Number of words such as “um,” “aah,” and “hmmm.” This feature can may indicate impaired lexical access, syntactic difficulties, discourse planning deficits
	False starts	Number of partial words. This feature measures deficits at phonetic-phonological, lexico-semantic or discourse level
**Lexico-semantic**	Noun rate	Total number of nouns divided by total number of words
	Verb rate	Total number of verbs divided by total number of words
	Pronoun rate	Total number of pronouns divided by total number of words
	Noun-verb ratio	Total number of nouns divided by total number of verbs
	Pronoun-noun ratio	Total number of pronouns divided by total number of nouns
	Closed-class words	Total number of closed class words (determiners, prepositions, pronouns, conjunctions) divided by total number of words
	Idea density	Sum of verbs, adjectives, adverbs, prepositions, conjunctions divided by total number of words
	Frequency	Frequency with which a word occurs in some corpus of natural language; it can be measured for each part of speech (i.e., verb frequency, noun frequency, etc.)
	Mean log frequency of nouns	Mean logarithmic frequency, in a corpus of natural language, of nouns
	Familiarity	Subjective rating of how familiar a word seems
	Closed-class words errors	Total number of errors (subdivided into omission, insertion, and substitution) divided by total number of closed-class words
	Repaired sequences	Sequences of one or more complete words, resulting redundant by subsequent repetitions, elaborations or alternative expressions. This feature measures deficits at: lexico-semantic, syntactic or discourse level
	Semantic errors	Total number of errors occurring when a target word is replaced by a term that could, from the context, be identified as a semantically related item; this feature includes: semantic (semantically erroneous substitutions) and visual paraphasias (substitutions that are visually similar to the target object)
	Word-finding difficulties	The proportion of speech comprising word-finding difficulties as indicated by a pause, an immediate repetition of a previous word or production of an indefinite term
	Indefinite terms	Total number of empty words without specific meaning; nonspecific nouns or pronouns (i.e., “whatever,” “something,” “stuff”) that made ambiguous or general reference
	Revision	The count of pause positions where the speaker retraces a preceding error and then make a correction. This feature measures deficits at: lexico-semantic or discourse level
	Perseveration	Total number of items appearing out of context after or before its appearance at the grammatically correct place. This feature measures deficits at: lexico-semantic or discourse level
	Repetitions	Total number of immediate word repetitions. This feature measures deficits at: lexico-semantic or discourse level
	Response to word finding delay	The most common response to word finding delays, that is whether patients appear unaware of their problem, produce an approximation of the target word or actively search and produce the target word. This feature measures deficits at: lexico-semantic or discourse level
**Morphosyntactic**	Inflectional errors	Total number of errors in conjugation and declination of words
**Syntactic**	Mean length of utterances	The average number of morphemes or words per utterance
	Utterances	Total number of utterances (a utterance represents any speech sequence consisting of one or more words and preceded and followed by silence)
	Clauses per sentences	The average number of clauses per sentence; a sentence is a grammatically complete string of words expressing a complete thought, a group of words that forms an independent grammatical unit
	Incomplete sentences	Total number of sentences that are abandoned after producing subjects and verbs. This feature measures deficits at: lexico-semantic level, syntactic level or discourse level
	Reduced sentences	Total number of subordinated sentences with nominal verb forms (which are either participles or gerund)
	Nouns with determiner	The proportion of nouns with determiner
	Well-formed sentences	Percentage of utterances that are well-formed sentences
	Verb phrases	Total number of phrases consisting of at least a verb and its dependents
	Embeddings	Total number of sentences embedded within other sentences
	Coordinate sentences	Total number of phrases united by a coordinating conjunction such as “and”
	Dependent clauses	Total number of clauses that does not form a sentence on its own
	Mean depth	Mean Yngve depth of each node in the parse tree, averaged over all sentences; Yngve depth of a sentence is the maximum number of items in the phrase structure tree that have to be stored during the construction of the sentence
	Total depth	Mean Yngve depth of each node in the parse tree, averaged over all sentences
	Syntactic errors	Erroneous uses of grammatical rules involving sentence structure or ungrammatical sentences
**Discourse and Pragmatic**	Total words	Total number of produced words
	Discourse markers	Total number of words or phrases that function primarily as a structuring unit of spoken language (i.e.: “you know,” “you see,” “well”)
	Cohesion	Number of utterances containing: Referential cohesion (correct pronominal reference) Temporal cohesion (correct use of verb tense) Causal cohesion (appropriate conjunctions)
	Correct pronoun	Correct pronominal reference
	Local coherence	The linkage of each event with the preceding event, which is accomplished by rhetorical markers such as sequencing adverbials, pronominal reference to preceding nouns, and statements of cause and effect
	Global coherence	A variable that registers whether the speaker acknowledges the point of the story
	Microproposition	Number of utterances which provide details given in addition to the central topic
	Implausible or irrelevant details	Total number of utterances which provide implausible or irrelevant information given in addition to the central topic
	Index of discourse effectiveness	The ratio of the total number of recalled words divided by the number of macropropositions or information contents
	Errors in content elements	Total number of utterances containing factually inaccurate elements
	Information content	Total number of the relevant, truthful, non-redundant utterances, excluding phrases containing indefinite terms and redundant words, inappropriate phrases and implausible details
	Information units	Total number of correct information units; information units are usually subdivided in subjects, places, objects, and actions (picture description) or in narrative sequences (story narration)
	Topic maintenance	Maintenance of the topic; total number of information content divided by total number of disruptive topic shifts
	Efficiency	Total number of information units divided by duration of speech sample (in seconds)

**Table 4:** **Comparisons between each neurodegenerative disease group and healthy controls for the most relevant features (please see text for details)**.

**Linguistic level**	**Linguistic feature**	**NF/av vs. HC**	**Sv vs. HC**	**L/Pv vs. HC**	**AD vs. HC**	**aMCI vs. HC^∧^**	**ALS vs. HC**	**PD vs. HC**	**PDD vs. HC**	**LBD vs. HC**	**CBS vs. HC^∧^**	**HD vs. HC^∧^**
**Phonetic and Phonological**	Speech rate	NF/av < HC^***^	Sv < HC^***^	L/Pv < HC^***^	AD < HC^***^	–	^*^	^*^	PDD < HC^***^	LBD < HC^***^	CBS < HC^**^	–
	No. of pauses	–	–	–	–	–	–	PD > HC^**^	–	–	–	–
	Between-utterance pause duration	–	–	–	–	–	–	PD > HC^**^	–	–	–	–
	Prosody: F0 SD	–	–	–	^*^	–	–	PD < HC^**^	–	–	–	–
	Prosody: Intensity SD	–	–	–	–	–	–	PD < HC^**^	–	–	–	–
	Hesitation ratio	NF/av > HC^**^	–	–	AD > HC^***^	–	–	–	–	–	–	–
	Phonemic errors	NF/av > HC^***^	^*^	L/Pv > HC^***^	^*^	–	^*^	^*^	PDD > HC^***^	LBD > HC^***^	–	–
	Total locution time	NF/av > HC^***^	^*^	^*^	^*^	^*^	^*^	^*^	^*^	^*^	–	–
	Filled pauses	^*^	^*^	L/Pv > HC^***^	^*^	–	^*^	–	–	–	–	–
	False starts	^*^	Sv > HC^***^	L/Pv > HC^**^	^*^	–	^*^	–	–	–	–	^*^
**Lexico-semantic**	Noun rate	^*^	Sv < HC^***^	^*^	^*^	–	^*^	^*^	^*^	^*^	–	–
	Pronoun rate	^*^	Sv > HC^***^	L/Pv > HC^**^	^*^	–	^*^	–	–	–	–	–
	Noun-verb ratio	^*^	Sv < HC^***^	–	–	–	–	–	–	–	–	–
	Pronoun-noun ratio	–	–	–	AD > HC^***^	–	–	–	–	–	–	–
	Closed-class words	^*^	^*^	^*^	AD > HC^***^	^*^	^*^	^*^	–	–	–	^*^
	Idea density	–	–	–	AD < HC^***^	–	–	–	–	–	–	–
	Frequency	^*^	Sv > HC^**^	–	AD > HC^***^	–	–	–	–	–	–	–
	Closed-class words errors	NF/av > HC^***^	^*^	–	^*^	–	–	–	–	–	–	–
	Repaired sequences	^*^	^*^	L/Pv > HC^***^	^*^	–	^*^	–	–	–	–	–
	Semantic errors	^*^	Sv > HC^***^	^*^	AD > HC^***^	–	ALS > HC^**^	–	–	–	–	–
	Word-finding difficulties	^*^	Sv > HC^**^	–	AD > HC^***^	–	–	–	–	–	–	^*^
	Indefinite terms	–	Sv > HC^**^	–	AD > HC^***^	–	–	–	–	–	–	–
	Revision	–	^*^	–	AD > HC^***^	–	–	^*^	^*^	^*^	–	^*^
	Perseveration	–	Sv > HC^**^	–	–	–	–	–	–	–	–	–
	Repetitions	–	–	–	AD > HC^***^	aMCI > HC^**^	–	–	–	–	–	–
	Response to word finding delay	–	–	–	AD < HC^***^	–	–	–	–	–	–	–
**Morpho-syntactic**	Inflectional errors	NF/av > HC^***^	Sv > HC^**^	–	AD > HC^***^	–	–	–	–	–	–	–
**Syntactic**	Mean length of utterances	NF/av < HC^***^	Sv < HC^***^	^*^	AD < HC^***^	–	ALS < HC^***^	^*^	^*^	^*^	–	–
	Utterances	^*^	^*^	^*^	^*^	–	ALS < HC^***^	^*^	PDD < HC^**^	LBD < HC^**^	–	^*^
	Incomplete sentences	NF/av > HC^***^	Sv > HC^**^	^*^	^*^	–	ALS > HC^**^	–	–	–	–	^*^
	Reduced sentences	–	Sv > HC^**^	–	AD > HC^***^	–	–	–	–	–	–	–
	Nouns with determiner	NF/av < HC^***^	^*^	^*^	^*^	–	–	–	–	–	–	–
	Well-formed sentences	^*^	Sv < HC^***^	L/Pv < HC^***^	^*^	–	ALS < HC^**^	^*^	PDD < HC^***^	LBD < HC^***^	–	HD < HC^**^
	Verb phrases	NF/av < HC^***^	^*^	–	AD < HC^**^	–	–	–	–	–	–	–
	Embeddings	NF/av < HC^***^	Sv > HC^**^	^*^	^*^	–	^*^	^*^	–	–	–	^*^
	Coordinate sentences	NF/av < HC^***^	^*^	–	^*^	–	–	–	–	–	–	–
	Dependent clauses	NF/av < HC^***^	^*^	^*^	^*^	–	–	^*^	^*^	LBD < HC^***^	–	^*^
	Mean depth	^*^	Sv < HC^***^	–	^*^	–	–	–	–	–	–	–
	Total depth	^*^	Sv < HC^***^	–	^*^	–	–	–	–	–	–	–
	Syntactic errors	NF/av > HC^***^	^*^	L/Pv > HC^**^	^*^	–	^*^	–	–	–	–	HD > HC^**^
**Discourse and Pragmatic**	Total words	NF/av < HC^***^	^*^	^*^	^*^	–	ALS < HC	^*^	PDD < HC	LBD < HC^***^	–	HD < HC^**^
	Discourse markers	^*^	^*^	–	AD > HC^***^	–	–	–	–	–	–	–
	Cohesion	NF/av < HC^**^	–	–	AD < HC^***^	^*^	–	–	–	–	–	–
	Correct pronoun	–	–	–	AD < HC^***^	–	–	–	–	–	–	–
	Local coherence	NF/av < HC^***^	Sv < HC^***^	L/Pv < HC^**^	AD < HC^***^	–	ALS < HC^**^	^*^	PDD < HC^***^	LBD < HC^***^	CBS < HC^**^	–
	Global coherence	^*^	^*^	L/Pv < HC^**^	AD < HC^***^	–	^*^	^*^	PDD < HC^***^	LBD < HC^***^	CBS < HC^**^	–
	Microproposition	–	–	–	AD > HC^**^	aMCI > HC^**^	–	–	–	–	–	–
	Implausible or irrelevant details	^*^	–	–	AD > HC^***^	aMCI > HC^**^	–	–	–	–	–	^*^
	Information content	NF/av < HC^**^	Sv < HC^**^	–	AD < HC^***^	–	ALS < HC^**^	–	–	–	CBS < HC^**^	–
	Information units	NF/av < HC^***^	Sv < HC^***^	L/Pv < HC^**^	AD < HC^***^	–	ALS < HC^***^	^*^	PDD < HC^**^	LBD < HC^**^	–	^*^
	Subjects	–	–	–	AD < HC^***^	–	–	–	–	–	–	^*^
	Actions	–	–	–	AD < HC^***^	–	–	–	–	–	–	HD < HC^**^
	Topic maintenance	NF/av < HC^***^	^*^	L/Pv < HC^**^	AD < HC^***^	–	^*^	^*^	PDD < HC^***^	LBD < HC^***^	CBS < HC^**^	–
	Efficiency	–	–	–	AD < HC^***^	–	–	–	–	–	–	–

HC, Healthy Controls; NF/Av, non-fluent variant of PPA; Sv, semantic variant of PPA; L/Pv, logopenic variant of PPA; AD, Alzheimer's Disease; aMCI, amnestic Mild Cognitive Impairment; ALS, Amyotrophic Lateral Sclerosis; PD, Parkinson's Disease; PDD, Parkinson's Disease with Dementia; LBD, Dementia with Lewy Body; CBS, Corticobasal Syndrome; HD, Huntington's Disease.

∧ , pathologies analyzed by less than three studies;

* , not relevant, i.e., attested in at least one study, but not found to be significant;

** , attested only in one study and reported as significant;

*** *, relevant, i.e., attested and reported as significant in at least more than half of the studies (with at least three studies investigating the same feature) or in two out of two studies; –, not attested*.

### Primary progressive aphasia

A total number of 15 papers were included, 12 about the non-fluent variant, 11 about the semantic variant, and only 3 about the logopenic variant. Only two studies report a comparison among the three variants. Table 4 reports a summary of the most relevant features for each variant and Table 5 shows a comparison among the three PPA variants. Results for the complete list of variables are reported in Supplementary Table 2.

**Table 5:** **Comparison among the three variants of the Primary Progressive Aphasia (PPA)**.

**Linguistic level**	**Linguistic feature**	**NF/Av vs. Sv**	**Sv vs. L/Pv**	**NF/Av vs. L/Pv**
Phonetic and phonological features	Mean first auto correlation function	NF/Av < Sv^**^	–	–
	Filled pauses	NF/av > Sv^**^	Sv < L/Pv^**^	^*^
	Speech rate	NF/Av < Sv^***^	Sv > L/Pv^***^	^*^
	Maximum speech rate	NF/Av < Sv^**^	Sv > L/Pv^**^	NF/Av < L/Pv^**^
Lexico-semantic features	Noun rate	^*^	^*^	NF/Av > L/Pv^**^
	Verb rate	^*^	^*^	NF/Av < L/Pv^**^
	Pronoun rate	NF/Av < Sv^**^	Sv > L/Pv^**^	NF/Av < L/Pv^**^
	Closed-class words	^*^	^*^	NF/Av < L/Pv^**^
	Mean log frequency of nouns	NF/Av < Sv^**^	Sv > L/Pv^**^	^*^
	Familiarity (nouns)	NF/Av < Sv^***^	–	–
	Repaired sequences	^*^	Sv < L/Pv^**^	^*^
**MORPHOSYNTACTIC FEATURES**				
Syntactic features	Clauses per sentences	NF/Av > Sv^**^	–	–
	Incomplete sentences	NF/Av < Sv^**^	^*^	^*^
	Embeddings	NF/Av < Sv^**^	Sv > L/Pv^**^	^*^
	Dependent clauses	NF/Av < Sv^***^	^*^	^*^
	Syntactic errors	^*^	Sv < L/Pv^**^	^*^
Discourse and pragmatic features	Total words	NF/Av < Sv^**^	^*^	NF/Av < L/Pv^**^
	Errors in content elements	NF/Av < Sv^**^	–	–

* , not relevant, i.e., attested in at least one study, but not found to be significant;

** , attested only in one study and reported as significant;

*** *, relevant, i.e., attested and reported as significant in at least more than half of the studies (with at least three studies investigating the same feature) or in two out of two studies; –, not attested; NF/Av, non-fluent/agrammatic variant of PPA; Sv, semantic variant of PPA; L/Pv, logopenic/phonological variant of PPA*.

#### Non-fluent variant

The majority of studies reports a lower speech rate in people suffering from the non-fluent variant of PPA than in non-brain-damaged persons. Several studies have documented that speech rate is less than one third of the rate of healthy seniors (Ash et al., 2009; Gunawardena et al., 2010; Wilson et al., 2010; Rogalski et al., 2011). In five out of six studies considered, patients' speech takes longer to be produced (low total locution time) and presents frequent phonemic errors. A greater number of phonetic errors in patients with the non-fluent variant with respect to non-brain-damaged persons has been reported only in one of the two studies.

An analysis of speech production at lexico-semantic level shows no differences between patients with non-fluent variant and non-brain-damaged persons. These patients, however, produce an increased number of errors in closed-class words (Knibb et al., 2009; Meteyard and Patterson, 2009; Sajjadi et al., 2012b), i.e., less nouns with determiners. At the syntactic level, an impoverished ability to generate complex syntactic structures is characterized by the low number of words per utterances, clauses, verb phrases, and coordinate sentences. In particular, a low number of embeddings is only reported in story narration and interview (Knibb et al., 2009; Fraser et al., 2014b), but not in the picture description task (Wilson et al., 2010), suggesting an inferior ability of the picture description task to detect syntactic impairment. An impairment at this level is also highlighted through the presence of frequent incomplete sentences and syntactic and inflectional errors (Graham et al., 2004; Sajjadi et al., 2012b).

The number of word produced by patients with non-fluent variant is consistently reduced when compared to non-brain-damaged persons. (Graham et al., 2004; Wilson et al., 2010; Ash et al., 2013; Fraser et al., 2014a; Ash and Grossman, 2015). In addition, these patients perform poorly on local coherence, showing a reduction of relevant information and difficulties in maintaining the topic (Ash et al., 2006; Sajjadi et al., 2012b; Ash and Grossman, 2015). In the description tasks, they have difficulties in achieving an accurate description of the scene represented in the pictures, providing only a few information units (Graham et al., 2004; Ash et al., 2006; Sajjadi et al., 2012b; Ash and Grossman, 2015).

#### Semantic variant

A lower speech rate associated with the presence of several false starts is reported in patients with the semantic variant when compared to non-brain damaged people (Ash et al., 2006, 2013; Meteyard and Patterson, 2009; Wilson et al., 2010; Sajjadi et al., 2012a; Fraser et al., 2014a; Ash and Grossman, 2015). These patients, however, produce a normal total number of words (but see Ash et al., 2013). No phonetic and phonemic errors are reported.

At the lexico-semantic level, patients with the semantic variant produce a reduced number of nouns (Ash et al., 2013; Fraser et al., 2014a; Jarrold et al., 2014; Ash and Grossman, 2015), often replaced by pronouns (Wilson et al., 2010; Jarrold et al., 2014), which are consistently in higher when number when compared to non-brain damaged persons. A short mean word length has been similarly attributed to the impaired availability of words, rather than to the difficulty with producing long words (Fraser et al., 2014a). Some studies report in addition an increased frequency of the nouns and verbs produced (Fraser et al., 2014a). As expected, semantic errors are constantly found in these patients (Meteyard and Patterson, 2009; Sajjadi et al., 2012a). Lexical/semantic disorders may contribute to the paucity of content, resulting in a reduction of information units (Ash et al., 2006; Sajjadi et al., 2012a; Ash and Grossman, 2015). An impairment of local coherence is also reported (Ash et al., 2006; Ash and Grossman, 2015) denoting difficulties in discourse planning. Syntactic processing is largely intact, but a detailed analysis shows a reduced proportion of well-formed sentences (Ash et al., 2006; Ash and Grossman, 2015), as well as a simplification of syntax denoted by a reduction of the mean length of utterance (Meteyard and Patterson, 2009; Wilson et al., 2010; Sajjadi et al., 2012a; Ash and Grossman, 2015; Fraser et al., 2015b), and a decrease of complexity of syntactic structure (Fraser et al., 2015b). A summary of the most relevant linguistic variables associated with the semantic variant is reported in Table 4.

#### Logopenic variant

While most scholars agree on the importance of connected speech analysis in identifying the logopenic variant, only three studies are available. In addition, only a few features at each linguistic level have been investigated (see Table 4). Typical changes are a low speech rate and an increased number of filled pauses and false starts (Wilson et al., 2010; Ash et al., 2013; Ash and Grossman, 2015). Phonemic errors are also considered as an important feature of this variant (Wilson et al., 2010; Ash et al., 2013; Ash and Grossman, 2015). At the lexico-semantic level, difficulties are highlighted by the increased number of repaired sequences (Wilson et al., 2010; Ash and Grossman, 2015). A reduced number of open class words (Ash et al., 2013) and an increased number of pronouns (Wilson et al., 2010) have also been reported. While the absence of a frank agrammatism was considered as a core diagnostic feature, an impairment at syntactic level may be suggested by the reduced proportion of well-formed sentences (Ash et al., 2013; Ash and Grossman, 2015). The presence of these heterogeneous features leads to a great difficulty in the characterization of the logopenic and non-fluent aphasias as two distinct variants (Mesulam et al., 2012; Sajjadi et al., 2014). Mild impairment at the discourse and pragmatic level is ascribed to the reduction of coherence (global and local), and to difficulties in maintaining the topic and in identifying the information units (Ash and Grossman, 2015).

#### Comparisons between PPA variants

Very few studies report a comparison among the different variants. A summary of these results is reported in Table 5. Several investigations indicate that the semantic variant can be easily differentiated from the other two variants through quantitative tests (naming and single word comprehension; Savage et al., 2013). Additional information can however be gained from connected speech analysis. According to the current criteria, persons with the non-fluent variant show a greater impairment at both phonetic/phonological and syntactic levels than people suffering from the semantic variant. Speech in the non-fluent variant is in fact characterized by a slow rate of speech (Ash et al., 2006, 2013; Wilson et al., 2010) and a greater number of filled pauses (Wilson et al., 2010). At the syntactic level the simplification of syntax is helpful in distinguishing the two variants, with people with non-fluent variant producing a reduced number of dependent clauses (Ash et al., 2013; Fraser et al., 2014a) compared to patients with the semantic variant, who typically produce nouns with higher values of familiarity (Wilson et al., 2010; Fraser et al., 2013, 2014a) than persons with the non-fluent variant.

Also people with the logopenic variant show a greater impairment at both phonological and syntactic levels when compared to patients with the semantic variant. The speech produced by these patients is in fact characterized by a reduction of speech rate associated with a greater number of filled pauses and repaired sequences. Syntactic errors and a reduced number of embeddings are also found in people suffering from logopenic variant when compared to those with semantic variant (Wilson et al., 2010), who produce more pronouns and high-frequency nouns than the logopenic variant.

The distinction of non-fluent variant form the logopenic variant remains the most debated issue. Information deriving from connected speech are precious but investigated only in very few studies. Difference are present at the lexico-semantic level, where patients with the non-fluent variant produce more nouns (Ash et al., 2013), whereas patients with the logopenic variant more function words, in particular pronouns, and verbs (Wilson et al., 2010). In addition, patients with the logopenic variant produce a greater number of words (Ash et al., 2013) than patients with non-fluent variant.

### Alzheimer's disease and amnestic mild cognitive impairment

A total number of 36 papers were included, 33 reporting data only for AD, and 3 for both aMCI and AD. Unfortunately, some of the studies do not report separately results according to different stages of disease. The AD group considered for this review includes mostly early and mild AD, but also studies conducted in more advanced stages of disease (Bucks et al., 2000; Forbes-McKay and Venneri, 2005; de Lira et al., 2011; Fraser et al., 2015a; Yancheva et al., 2015). This aspect is detailed in Supplementary Table 3.

#### Alzheimer's disease

The majority of studies using connected speech have investigated lexico-semantic and discourse-pragmatic levels (respectively, 80 and 77.5%) followed by syntactic (57.5%), phonetic and phonemic (55%), and morphological domains (35%). All the results are reported in the Supplementary Table 3.

At phonetic and phonological level, speech in patients with AD is principally characterized by a low speech rate and by frequent hesitations (Hoffmann et al., 2010; Sajjadi et al., 2012a). Further altered variables, which have been rarely investigated, include acoustic measures (see Supplementary Table 3). Phonemic errors occur very rarely (2/12).

At the lexico-semantic level, the analysis of part of speech variables reveals that AD patients produce a greater number of closed class words (Croisile et al., 1996; Sajjadi et al., 2012a; Drummond et al., 2015), in particular more pronouns than non-brain damaged persons (Ahmed et al., 2013b; Jarrold et al., 2014), denoting low content density. In the picture description task, it is possible to detect compensatory deictic use, with patients with AD having a tendency for spatial deictic overuse, determined by the properties of the communicative contexts (Nicholas et al., 1985; March et al., 2006). This result is not attested in the story narration task, where an underuse of person deictics is reported (March et al., 2006). People suffering from AD produce more high-frequency words as well as semantic and lexical errors (Kempler et al., 1987; Kavé and Levy, 2003), such as word finding difficulties (Croisile et al., 1996; Forbes-McKay and Venneri, 2005; Ash et al., 2007; de Lira et al., 2011; Forbes-McKay et al., 2013), indefinite terms (Nicholas et al., 1985; Feyereisen et al., 2007; Visch-Brink et al., 2009; Lai, 2014), revision (Forbes-McKay and Venneri, 2005; Forbes-McKay et al., 2013; Orimaye et al., 2014), repetitions (Nicholas et al., 1985; Visch-Brink et al., 2009; de Lira et al., 2011; Guinn and Habash, 2012; Sajjadi et al., 2012a; Orimaye et al., 2014; Drummond et al., 2015), and neologisms (Fraser et al., 2015a; Yancheva et al., 2015) than healthy persons.

At the morpho-syntactic level, a greater number of inflectional errors in person with AD than in non-brain damaged persons is the only relatively consistent result, reported in two out of three studies (Altmann et al., 2001; Cuetos et al., 2007; Sajjadi et al., 2012a). Other features in this domain have been rarely or inconsistently reported across studies; see Supplementary Table 3 for a complete list. Although, several studies agree in considering syntactic processes preserved in persons with AD (Kavé and Levy, 2003; Forbes-McKay and Venneri, 2005), a consistent number of the studies included in this review report a simplification of syntax, characterized by reduced sentences and short utterances (Ash et al., 2007; de Lira et al., 2011; Sajjadi et al., 2012a; Orimaye et al., 2014; Ash and Grossman, 2015; Yancheva et al., 2015).

Discourse and pragmatic levels are impaired in patients with AD according to a number of studies (Carlomagno et al., 2005; Sajjadi et al., 2012a; Ahmed et al., 2013a; Lai, 2014; Ash and Grossman, 2015; Drummond et al., 2015). A greater number of discourse markers (Visch-Brink et al., 2009) is reported in persons with AD when compared to non-brain damaged persons, particularly in the case of interviews, and probably reflecting lexico-semantic deficits (Hoffmann et al., 2010). Common errors at the discourse level concern referential cohesion, with an inadequate or ambiguous use of pronouns to characterize the antecedent (Nicholas et al., 1985; Ripich et al., 2000; Altmann et al., 2001; Dijkstra et al., 2004; Lai, 2014; Drummond et al., 2015). Additionally, Dijkstra et al. (2004) report a deficit in temporal cohesion. Discourse in patients with AD is characterized by compromised coherence, with many of irrelevant and implausible details. A low efficiency and the failure in mentioning key concepts, namely mentioning fewer persons, objects, and actions than healthy persons (Bschor et al., 2001; Kavé and Levy, 2003; Ahmed et al., 2013a), leads to uninformative picture descriptions in these patients (Nicholas et al., 1985; Croisile et al., 1996; Shimada et al., 1998; Carlomagno et al., 2005; Cuetos et al., 2007; Feyereisen et al., 2007; Ahmed et al., 2013a,b; Forbes-McKay et al., 2013; Lai, 2014; Fraser et al., 2015a).

#### Amnestic mild cognitive impairment

The phonetic and phonological levels, investigated in only two studies, are not affected in aMCI. Their speech does not present alteration at lexico-semantic level. Only one study reports more repetitions than non-brain damaged persons in a story narration task (Drummond et al., 2015).

The discourse and pragmatic level seems to be the most affected in persons with aMCI. Drummond et al. (2015) have identified the coherence as a relevant measure to discriminate non-brain damaged persons from persons with aMCI, with aMCI producing a less informative discourse, presenting more implausible/irrelevant details and incomplete content elements, but with an exhaustive number of information unit (Drummond et al., 2015).

#### Comparison between AD and aMCI

Ahmed et al. (2013b) have explored part of speech variables comparing patients with AD and patients with aMCI, identifying a higher number of pronouns in AD. Discourse in aMCI seems more efficient, coherent and informative than in the case of patients with AD (for a summary please see Table 6).

**Table 6:** **Comparison between Alzheimer's disease and amnestic Mild Cognitive Impairment**.

**Linguistic level**	**Linguistic feature**	**AD vs. aMCI**
Phonetic and phonological features		–
Lexico-semantic features	Pronoun rate	AD > aMCI^**^
	Idea density	AD < aMCI^**^
Morphosyntactic features		–
Syntactic features		–
Discourse and pragmatic features	Information content	AD < aMCI^**^
	Index of discourse effectiveness	AD < aMCI^**^
	Information units	^*^
	Efficiency	AD < aMCI^**^

* , not relevant, i.e., attested in at least one study, but not found to be significant;

** *, attested only in one study and reported as significant; –, not attested; AD, Alzheimer's Disease; aMCI, amnestic Mild Cognitive Impairment*.

### Movement disorders

Only a few studies have investigated connected speech in movement disorders; one study in PD, one in CBS, and one in HD; four studies compared PD, PDD, and LBD performances, and one HD with PD. Most studies using connected speech investigated lexico-semantic (78%), discourse-pragmatic (66%), and syntactic levels (66%), followed by phonetic (55%) and phonemic and morphological domains (33%). Schematic results for the most relevant features can be found in Table 4; detailed results are reported in Supplementary Table 4.

#### Parkinson's disease

Despite the paucity of studies focusing on connected speech in PD, it is possible to indicate that the phonetic impairment in patients with PD is mainly detected by variables related to the duration of pauses (Rusz et al., 2011; Ash et al., 2012b) and by the alteration of prosody (Rusz et al., 2011), rather than speech rate or phonetic errors. No significant differences have been found between patients and non-brain damaged people at the phonological, lexico-semantic, morpho-syntactic, and syntactic levels. Inconsistent results are reported at the discourse and pragmatic level. Only a study (Ash et al., 2012a) out of three revealed a reduction of local coherence and topic maintenance for PD patients. Overall, these findings indicate that the language production of PD patients is basically intact, with the exception of the phonetic/acoustic level.

#### Parkinson's disease with dementia and dementia with lewy body

PDD and LBD patients exhibit almost overlapping patterns of impairment during speech production when evaluated with story narration tasks (Ash et al., 2011, 2012a,b; Ash and Grossman, 2015). The deficits encompass all the linguistic levels, with the exception of the lexico-semantic domain. Phonetic features, such as speech rate and phonetic errors are abnormal (Ash et al., 2011, 2012b; Ash and Grossman, 2015) and the dysfunction is at least partly related to motor impairment. PDD and LBD patients show also high rates of phonemic errors (Ash et al., 2011; Ash and Grossman, 2015). At the syntax level, these patients obtain lower performances compared to non-brain damaged persons on features addressing the number of well-formed sentences (Ash et al., 2011, 2012b; Ash and Grossman, 2015), while the number of dependent clauses is reduced only in persons with LBD (Ash et al., 2011, 2012b; Ash and Grossman, 2015). Interestingly, a single study observes that persons with LBD present more grammatical errors than non-brain damaged persons and PDD (Ash et al., 2012b). Alterations are also described at the discourse-pragmatic level. PDD and LBD patients are impaired relative to healthy speakers on all measures of narrative organization (Ash et al., 2011, 2012a; Ash and Grossman, 2015). They manifest difficulty in connecting one event to the next, maintaining the theme and in understanding the story (as reflected by the low level of global and local coherence and topic maintenance, Ash et al., 2011, 2012a; Ash and Grossman, 2015).

#### Corticobasal syndrome

To the best of our knowledge, only one study analyzed connected speech in persons with CBS, focusing on discourse and pragmatic level. Narrative discourse is significantly impaired in CBS when compared to non-brain damaged persons, and the performance is related to atrophy in the right parietal lobe and bilateral dorsolateral prefrontal cortex (Gross et al., 2010). Discourse in CBS patients is less accurate than in controls and presents lower global and local coherence and topic maintenance relative to healthy speakers, suggesting that CBS patients have difficulty in maintaining the story theme and in connecting events, with the majority of patients failing to identify the overall point of the story. These deficits cannot be entirely accounted by troubles in perceiving and naming elements of scenes or by the inability to remember story events.

#### Huntington's disease

Speech deficits in HD patients have been investigated by two studies (Murray and Lenz, 2001; Jensen et al., 2006), and only a few linguistic variables have been investigated at each level. With a consensus across studies, HD patients are unimpaired at the lexico-semantic level and, although with less consistency, at the phonological domain. Deficits are reported in the phonetic (reduced speech rate), syntactic and discourse levels, whereas morpho-syntactic features have not been investigated. At the syntactic level, HD patients produce high rates of syntactic errors (Jensen et al., 2006), reduced number of utterances and of well-formed sentences (Murray and Lenz, 2001), without a reduction of syntactic complexity, measured with the number of dependent clauses. These results are consistent with the spontaneous speech reduction and simple sentence construction evidenced by interviews (Podoll et al., 1988; Murray and Lenz, 2001). At the discourse level, HD patients provide as much comments and information about the target picture as non-brain damaged persons, but fewer action information units, suggesting that a deficit of information content specific to action (Jensen et al., 2006).

#### Comparison between parkinson's disease, parkinson's disease with dementia, and dementia with lewy body

Phonetic and phonemic errors are more frequent in PDD and LBD patients than in persons with PD (Ash et al., 2011, 2012b; Ash and Grossman, 2015), and have been related mainly to executive functions, rather than to motor difficulties. Similarly, the grammatical expression, characterized by a reduction of well-formed sentences and of dependent clauses, is significantly compromised in persons with PDD and LBD, but not in non-demented PD patients (Ash et al., 2011, 2012b; Ash and Grossman, 2015). Speech rate appears to be useful in distinguishing PDD from LBD, with persons with PDD being less impaired and producing a higher average of words per minute (Ash et al., 2011, 2012b). Although, only a small number of studies investigated the discourse-pragmatic level (Ash et al., 2011, 2012a; Ash and Grossman, 2015), narrative organization is impaired in both LBD and PDD patients, with LBD exhibiting higher difficulties than PDD in local coherence and topic maintenance. These results are summarized in Table 7.

**Table 7:** **Comparison among Parkinson's Disease, Parkinson's Disease with Dementia and Dementia with Lewy Body**.

**Linguistic level**	**Linguistic feature**	**PDD vs. LBD**	**PD vs. PDD**	**PD vs. LBD**
Phonetic and Phonological features	Speech rate	PDD > LBD^***^	PD > PDD^**^	PD > LBD^**^
	Standardize phonation time	PDD > LBD^**^	–	–
	Phonetic-Phonemic errors	–	PD < PDD^**^	PD < LBD^**^
Lexico-semantic features	–			
Morphosyntactic features	–			
Syntactic features	Well-formed sentences	PDD > LBD^**^	PD > PDD^**^	PD > LBD^***^
	Dependent clauses	PDD > LBD^**^	–	PD > LBD^**^
Discourse and Pragmatic features	Total words	–	PD > PDD^**^	PD > LBD^**^
	Local coherence	PDD > LBD^**^	–	–
	Topic maintenance	PDD > LBD^**^	–	–

** , attested only in one study and reported as significant;

*** *, relevant, i.e., attested and reported as significant in at least more than half of the studies (with at least three studies investigating the same feature) or in two out of two studies; –, not attested; PD, Parkinson's Disease; PDD, Parkinson's Disease with Dementia; LBD, Dementia with Lewy Body*.

### Amyotrophic lateral sclerosis

Only four studies analyzed connected speech in persons with ALS, using picture description and story narration, with a major focus on phonetic/acoustic, syntactic and discourse-pragmatic levels. The results are reported in the Supplementary Materials (Supplementary Table 5). Relevant linguistic variables are summarized in Table 4. Only one study analyzed phonetic and semantic levels (Tsermentseli et al., 2016) evidencing that these language measures are unable to predict group membership (i.e., ALS vs. non-brain damaged persons). On the contrary, syntactic and pragmatic impairments have been reported in ALS. Syntactic processing impairments are detected in the form of a reduced number of utterances, with shorter sentences in persons with ALS when compared to healthy speakers (Roberts-South et al., 2012; Ash et al., 2015; Tsermentseli et al., 2016). Discourse and pragmatic impairment is mainly characterized by a reduced number of words (Ash et al., 2014, 2015; Tsermentseli et al., 2016), whereas information units and the content of discourse produced are less informative (Roberts-South et al., 2012). Additionally, ALS patients have difficulties in connecting one event to the next (local coherence) and in maintaining the theme of the story (search theme; Ash et al., 2014).

## Discussion

Language assessment has an important role in the clinical diagnosis of neurodegenerative diseases, not only in the conditions where language disorders are a central feature, but also in those in which language impairment is less evident, and sometimes obscured by other cognitive and non-cognitive features.

A central component of any comprehensive evaluation of language is the analysis of connected speech, a simple procedure allowing to collect large amounts of data (Peintner et al., 2008), which can be considered as an ecologically valid method of monitoring language changes (Arkin and Mahendra, 2001) for its closeness to the natural communication exchange. This stands in contrast with most of the traditional language tasks based on single word or isolated sentence processing. All levels of language organization, including phonetic, phonological, lexico-semantic, morpho-syntactic, and pragmatic processing, can be analyzed from connected speech.

In this review, we revised the studies focusing on connected speech in the most prevalent neurodegenerative diseases, including PPA, Alzheimer's disease, movement disorders, and ALS. We reviewed the tasks used to elicit connected speech and provided a classification of the multiple linguistic variables that can be extrapolated. Finally, we identified the linguistic variables most frequently impaired in each linguistic domain and for each specific neurodegenerative disease, highlighting those useful for differential diagnosis.

### Task used to elicit speech samples

Picture description, story narration, and different modalities of conversation/interview are the most common tasks for collecting connected speech. These tasks encourage participants to engage in an extended language production, similar to what happens in everyday life. On the basis of their intrinsic structure, the tasks appear to differ in sensitivity to damage to linguistic levels. Garrard and Forsyth (2010) suggested that the picture description leads to the production of simplified syntactic structures and that anomia can be more easily compensated when we talk freely, as in the interview, than in the picture description task. Similarly, March et al. (2006) found that picture description encourages the use of nouns and deixis. Studies focusing on discourse and pragmatic (coherence, cohesion, etc.) variables, typically adopt story narration and interviews rather than the picture description task (Dijkstra et al., 2004; Gross et al., 2010; Ash et al., 2011, 2012a,b; Lai, 2014; Ash and Grossman, 2015; Drummond et al., 2015).

The evidence supporting these claims is however limited: only three studies have targeted neurodegenerative diseases with incomplete agreement (Sajjadi et al., 2012a,b; Ash et al., 2013). The picture description task is reported as more suitable in detecting lexico-semantic disorders, while the semi-structure interview was more sensitive in revealing impairment affecting the morphological, syntactic, and discourse levels (Sajjadi et al., 2012a,b). A third study however reported that the story narration and the picture description can be used interchangeably (Ash et al., 2013). These discrepancies can be probably disentangled considering differences in the linguistic variables used for the analyses, the pathological samples, the tasks, and the statistical analyses. In particular, Ash et al. (2013) have investigated the phonetic and phonological level (speech rate, phonetic/phonemic errors, filled pauses), lexico-semantic domain (noun rate, open class words), syntactic level (mean length of utterances, well-formed sentences, dependent clauses), and one discourse variable (total words), using only a few variables for each level. Sajjadi et al. (2012a,b) have explored all linguistic domains in more details: phonetic and phonological (speech rate, hesitations, phonemic errors, false starts), lexico-semantic (open class word and closed class words errors, semantic errors, repaired sequences, circumlocutory comments), morpho-syntactic (inflectional errors, mean length of utterance, incomplete sentences, dependent clauses), discourse and pragmatic (discourse markers, information content, information units, spontaneity). In addition, Sajjadi et al. (2012a,b) used a paired *T*-test (or its nonparametric equivalent) to compare each linguistic variable of the two speech elicitation methods, considering each group separately. Ash et al. (2013), in contrast, used a correlation analysis considering all the subjects together.

The evidence is thus inconclusive and further studies, comparing the same tasks in different patient populations, are needed to identify the most suitable tasks for specific aims. Moreover, the relationship between tasks eliciting connected speech and traditional language tests (such as verbal fluency and picture naming) needs to be further investigated.

### Definitions and classification of the linguistic variables

Our review has shown a great heterogeneity of the linguistic variables used for connected speech analysis, as well as in the way in which these variables are measured and classified both in patients and in healthy subjects. Comparisons and generalization of the results are not easy. Any attempt to classify linguistic features has advantages and limitations. While the results may be too narrow to account for the clinical heterogeneity as well as the nature of a specific deficit, a standardization is needed in order to reduce differences in the categorization across studies, encouraging comparisons.

In this review, we adopt a descriptive classification across five different linguistic domains, from sound to discourse, according to traditional linguistic subdivisions. We define the level at which the linguistic variable is situated, i.e., sound or phoneme (for the phonetic and phonological level), word (for lexico-semantic level), sentence (for the morphological and syntactic level), and discourse (for the pragmatic level). This purely descriptive classification does not attempt to account for the nature of the alterations. In several cases, a single variable may be in fact caused by a deficit at different linguistic domains. False starts, for example, have been included among phonetic and phonological features, although this feature may also indicate an impairment at lexico-semantic level. An incorrect use of verbal tense, which has been classified at the morpho-syntactic level, may also reflect impairment at discourse level. Incomplete sentences, for example, have been attested in both non-fluent and semantic variants of PPA. These results could reflect different types of deficit: in the non-fluent variant, it could be the result of agrammatism; in the semantic variant, it could derive from difficulties at lexico-semantic level. Hints about the nature of language deficit are provided by the analysis of the overall performance profile of the patient, considering in addition all the linguistic variables derived from connected speech. The pattern associated with the semantic variant of PPA or Alzheimer's disease, which typically share lexico-semantic impairments, could include variables belonging to all the descriptive levels we have used. These patients may present, when compared with healthy subjects, a reduced speech rate and false starts (phonetic and phonological level); semantic errors (lexico-semantic level); reduced sentences (morphological and syntactic level); few information units (discourse-pragmatic level). All these alterations may be the consequence of an underlying lexico-semantic impairment (see Table 4).

The heterogeneity in classifying linguistic features may also account for divergences in the results obtained across studies. Features belonging to different linguistic domains have been sometimes grouped to form composite measures. Fluency, for instance, has been frequently used across studies in different combination of linguistic variables, including incomplete sentences (syntactic level), speech rate/hesitations (phonetic level), and discourse markers (discourse level). The measure of fluency has played a very important role in the classification of vascular aphasia (Goodglass et al., 1964), but has probably been a source of confusion if applied to a largely different set of conditions, such as the PPA spectrum (see discussion in Gorno-Tempini et al., 2008).

### Linguistic profiles of the main clinical conditions

This review suggests that connected speech is a valuable tool for the investigation of language disorders in neurodegenerative diseases, helping in clinical diagnosis.

The available evidence is supporting the crucial role of the analysis of connected speech for the differential diagnosis of PPA variants (Gorno-Tempini et al., 2011). It is particularly useful, with respect to traditional language tests, in aiding the distinction of the logopenic variant from the non-fluent one. One crucial issue is the distinction between motor speech disorders, typical of the non-fluent/agrammatic variant, and the phonological impairment of the logopenic variant. This distinction, as well as the diagnosis of agrammatism, is extremely difficult, if at all possible, on the basis of the traditional language production tasks, such as naming or repetition (see Savage et al., 2013 for an attempt in this direction). There is no doubt that the classification of errors at the phonetic/phonological level is a complex issue (Knibb et al., 2009; Sajjadi et al., 2012b). In the majority of the cases, it is very difficult disambiguating between phonetic and phonological errors on the basis of perceptual analysis, leading some authors to avoid the distinction. Some studies, however, explicitly reported this distinction (Wilson et al., 2010; Ash et al., 2013), or tried to isolate phonetic errors. Considering the latter variable together with additional variables, which can be only analyzed in connected speech, such as presence of hesitations, false starts, filled pauses, reduced maximum speech rate, and total locution time, can provide helpful cues for the differential diagnosis (see Tables 4, 5 and Supplementary Table 2).

Morphological and syntactic variables are crucial for the diagnosis of agrammatism in production. While some test can be useful for the diagnosis (Weintraub et al., 2009; De Leon et al., 2012), the analysis of connected speech yields a large amount of useful information to this aim (see Table 4 and Supplementary Table 2).

Finally, lexico-semantic impairments can be easily detectable using conventional single word tasks (see, for example, Hodges et al., 2008) and are easily observed in connected speech: for example, patients with the non-fluent variant produce more nouns (Ash et al., 2013), whereas patients with the logopenic variant use more function words, in particular pronouns, verbs (Wilson et al., 2010), and number of words (Ash et al., 2013). Other measures, assessing comprehension and non-verbal semantics (Catricalà et al., 2013), allow to recognize differences among the three variants.

In the case of AD, the lexico-semantic variables appear to be the most useful for the diagnosis. Several studies have shown mild lexico-semantic impairments also in the early or prodromal stages, such as in the case of aMCI, using semantic fluency (Joubert et al., 2010; for a review see Gainotti et al., 2014). Inconsistent results are shown by studies using object naming task (i.e., Ahmed et al., 2008; Joubert et al., 2010; but see Adlam et al., 2006; Balthazar et al., 2008; Clague et al., 2011; Choi et al., 2013; Gardini et al., 2013; Catricalà et al., 2015b); while data from naming pictures of unique entities including famous people, famous buildings and famous public events are more consistent (Estévez-González et al., 2004; Ahmed et al., 2008; Joubert et al., 2010; Clague et al., 2011; Gardini et al., 2013). Connected speech analysis appears to be less sensitive than these tasks in detecting subtle lexico-semantic in persons with aMCI when compared to healthy persons. Discourse and pragmatic level analysis seems instead to be promising for early diagnosis in persons with aMCI. However, it is important to note that very few studies (two) have been conducted. In the case of AD, global coherence and topic maintenance were reported to be more affected than in the semantic variant.

The analysis of connected speech in PD indicates that language is largely spared, with the exception of the phonetic/acoustic level, characterized by several pauses and impaired prosody, related to a disruption of the motor speech. The notion of impaired pragmatic abilities has not been consistently supported (Monetta and Pell, 2007; Monetta et al., 2009; Assal and Ghika, 2013). Several studies reported difficulties with verbs in PD, in particular with respect to verb inflection (Ullman et al., 1997) and verb generation (Péran et al., 2003; Crescentini et al., 2008). Connected speech analyses do not support these observations. Performance in verb inflection has been tested by two studies, and found to be unimpaired (Murray and Lenz, 2001; Ash and Grossman, 2015).

Connected speech appears to be helpful in distinguishing patients with PDD and LBD from pure PD, with syntactic and discourse domains more impaired in LBD and PDD. The results suggest that the occurrence of dementia is associated to reduced syntactic complexity, difficulty in connecting one event to the next, in maintaining the theme and in understanding the story, which are not found in PD (Ash et al., 2011, 2012a; Ash and Grossman, 2015). Noteworthy, pragmatic impairments appear to depend largely on a higher-level organizational component of the narrative, linked to executive control abilities (Ash et al., 2012a).

Coherence and topic maintenance at discourse level, as well as well-formed sentences and dependent clauses at syntactic level, are better preserved in PDD than in LBD (Supplementary Table 3). This observation is in line with the findings of a poorer executive functioning in mild LBD than in mild PDD (Aarsland et al., 2003).

Only one study analyzed connected speech in CBS (Gross et al., 2010) focusing uniquely on the discourse and pragmatic domains and finding an impairment at this linguistic level.

The syntactic and pragmatic domains are affected in HD, with a relative sparing of the other levels, confirming an impairment in language rule application due to striatum involvement. No firm conclusion can however be reached, since only two studies have addressed this issue assessing only a few linguistic variables (Murray and Lenz, 2001; Jensen et al., 2006).

The most predominant language impairment, emerging from the analyses of spontaneous speech of ALS patients, involves syntactic and pragmatic domains. Syntactic processing deficits are mainly described in the form of a simplification of syntax. This confirms the view that language dysfunction is an important aspect of the cognitive profiles of non-demented ALS patients (Taylor et al., 2013) and suggests that syntactic processing could be a marker for cognitive impairment. The results concerning pragmatic deficits of ALS patients are well documented but their nature is still controversial. Pragmatic impairment has been related at least in part to executive dysfunction, which is a common feature of ALS patients, and linked to the involvement of prefrontal regions (Ash et al., 2014). A recent study highlights a relative independence of pragmatic from executive domain in ALS (Bambini et al., 2016). These findings shed light on pragmatic impairment as a possible additional dimension of ALS deserving further consideration. Finally, according to recent studies (Leslie et al., 2015), subclinical semantic deficits might also occur in non-demented ALS patients. Accordingly, despite the paucity of studies analysing this linguistic level using connected speech, the only study accounting for this domain documented a greater number of semantic errors (Tsermentseli et al., 2016).

## Conclusions

The present review shows a detailed state of the art of the linguistic variables, extrapolated from connected speech analyses, depicting the linguistic profile of the most prevalent neurodegenerative diseases. This analysis should be useful in guiding clinicians in identifying and characterizing language disorders and in stimulating further research.

A final point worth considering is that, although the elicitation of spontaneous speech is a simple procedure, the quantitative and qualitative measurement of variables is a time consuming and difficult task, requiring considerable expertise. The recent and impressive progresses in computational linguistics are particularly promising, as they may ultimately lead to the development of powerful tools able to automatize most of the speech analysis processes, as well as the classification and clusterization of the productions. Of the articles reviewed here, 19 have analyzed data mixing automated and manual approaches. In these studies transcription and segmentation of the speech have mostly been made manually. Annotation and analysis of the text have been made using automated tools (i.e., part of speech tagger, parser), and machine learning methodology (support vector machine classifier, Bayesian networks, etc.). Machine Learning algorithms have been used to create diagnostic models using linguistic features resulting from speech samples (Garrard et al., 2014; Peintner et al., 2008; Jarrold et al., 2010, 2014; Guinn and Habash, 2012; Fraser et al., 2013, 2014a,b, 2015a,b; Rentoumi et al., 2014). In a few cases, the authors have also employed software for text analysis, namely the Linguistic Inquiry and Word Count, a tool that computes the frequency of words from predefined lists based on different categories such as psychological processes (i.e., emotional or cognitive), linguistic dimensions (i.e., articles, negations), or relativity (in time and space; Peintner et al., 2008; Jarrold et al., 2010, 2014). The development of this research area will be surely critical for both theoretical and clinical purposes.

## Author contributions

VB contributed to the design of the study, reviewed studies, interpreted data, and wrote the manuscript. EC contributed to the design of the study, interpreted data, wrote and revised the manuscript critically. MC contributed to the interpretation of data, wrote and revised the manuscript critically. CC contributed to the interpretation of data and revised the manuscript critically. AM gave an important intellectual content and revised the manuscript critically. SC supervised the development of the work and contributed in data interpretation, gave important intellectual content, revised the manuscript critically and acted as the corresponding author. All authors gave the final approval of the version to be published; they are accountable for all aspects of the work and ensure that questions related to the accuracy or integrity of any part of the work are appropriately investigated and resolved.

### Conflict of interest statement

The authors declare that the research was conducted in the absence of any commercial or financial relationships that could be construed as a potential conflict of interest.
