# Supplementary material for: Connected Speech in Neurodegenerative Language Disorders: A Review
Source: Front Psychol. 2017 Mar 6;8:269. doi: 10.3389/fpsyg.2017.00269 (PMC5337522; doi:10.3389/fpsyg.2017.00269)
Supplement: Supplementary file 1 [file Table1.docx]

Supplementary Material

Connected speech in neurodegenerative language disorders: a review

**Veronica Boschi^1^, Eleonora Catricalà^1^, Monica Consonni^2^, Cristiano Chesi^1^, Andrea Moro^1^, Stefano F. Cappa^1*^**

^1^ Institute for Advanced Study-IUSS Pavia, Palazzo del Broletto – Piazza Vittoria 15, 27100 Pavia, Italy

^2^ 3rd Neurology Unit and Motor Neuron Diseases Center, IRCCS Foundation “Carlo Besta” Neurological Institute, via Celoria 11, 20133 Milan, Italy

*** Correspondence:**Prof. Stefano F. Cappa
stefano.cappa@iusspavia.it

Supplementary Table 1. List of the 120 variables found in the literature, their definitions and synonymous, and their possible causes.

Legend: # = Total number

|  | Linguistic level and variables | Definition/how to measure | Synonymous | Possible causes/underlying deficit |
| --- | --- | --- | --- | --- |
|  | **Phonetic and phonological variables** | |  |  |
|  | **Mean first autocorrelation function** | The cross-correlation of a signal with itself at different points in time |  |  |
|  | **Skewness (MFCC)** | Mel-frequency cepstral coefficient (MFCC) measuring the lack of symmetry associated with tense or creaky voice |  |  |
|  | **Kurtosis (MFCC)** | Mel-frequency cepstral coefficient (MFCC) measuring the peakedness of the signal |  |  |
|  | **Number of pauses** | # produced pauses |  | -difficulty in articulation,  -impaired lexical access,  -syntactic difficulties,  -discourse planning deficits |
|  | **Total pause time** | Total duration (in seconds) of all pauses (minimum 1-second duration) per sample |  |  |
|  | **Mean pause duration** | Mean length of pauses > 0.15 ms |  |  |
|  | **Between –utterance pause duration** | Proportion of total duration of pauses between utterances |  | -syntactic difficulties  -discourse planning deficits |
|  | **Hesitation ratio** | Total duration of hesitations divided by the total speech time; an hesitation is defined as the absence of speech lasting more than 30 ms |  |  |
|  | **Filled pauses** | # Words such as “um”, “aah” and “hmmm” | - hesitation markers | -impaired lexical access,  -syntactic difficulties,  -discourse planning deficits. |
|  | **Vowel and consonant duration** | Duration of vowel and consonant |  |  |
|  | **Speech tempo** | Phonemes per second (with hesitations) |  |  |
|  | **Syllables per minutes** | Proportion of syllables produced per minutes |  |  |
|  | **Speech rate** | # words per minute |  |  |
|  | **Maximum speech rate** | Average of the words per minute for the three most rapid sequences of 10 or more words |  |  |
|  | **Total phonation time** | Total length of all non-silent segments divided by the number of words per minute (not including pauses) |  |  |
|  | **Standardized phonation time** | Text length divided by the total phonation time |  |  |
|  | **Total Locution Time** | The amount of time in the sample containing both speech and pauses |  |  |
|  | **F0 Standard Deviation** (prosody) | Variations of fundamental frequency, vibration rate of vocal folds |  |  |
|  | **Intensity Standard Deviation** (prosody) | Variations of average squared amplitude within a predefined time segment (“energy”) after removing any silence period exceeding 60 ms |  |  |
|  | **Prosodic disruption** | Disruption in the appropriate use of intonational contour, including alterations in pitch, volume and duration | - melodic line breakdown |  |
|  | **Phonetic errors** | Motor speech errors such as misarticulation not involving frank phonemic substitutions | - articulation errors  - distortions |  |
|  | **Phonemic errors** | Well-articulated phoneme substitutions, additions, and deletions | - phonological errors  - phonological paraphasias |  |
|  | **False starts** | # partial words |  | -deficits at phonetic-phonological level,  -impaired lexical access,  -syntactic difficulties,  -discourse planning deficits |
| Lexico-semantic features | | | | |
|  | **Noun rate** | # nouns divided by # words |  |  |
|  | **Verb rate** | # verbs divided by # words |  |  |
|  | **Demonstrative rate** | # demonstratives divided by # words |  |  |
|  | **Adjective rate** | # adjectives divided by # words |  |  |
|  | **Pronoun rate** | # pronouns divided by # words |  |  |
|  | **Adverb rate** | # adverbs divided by # words |  |  |
|  | **Conjunction rate** | # conjunctions divided by # words |  |  |
|  | **Possessive rate** | adjectives plus # pronouns divided by # words |  |  |
|  | **Noun-verb ratio** | # nouns divided by # verbs |  |  |
|  | **Noun ratio** | # nouns divided by (# nouns + # verbs) |  |  |
|  | **Pronoun-noun ratio** | # pronouns divided by # nouns |  |  |
|  | **Closed-class words** | # closed class words ( # determiners , # prepositions , # pronouns , # conjunctions) divided by # words |  |  |
|  | **Open class words** | # open class words (# nouns , # verbs, # adjectives , # adverbs) divided by # words |  |  |
|  | **Content Density** | # open class words divided by # closed class words |  |  |
|  | **Idea density** | Sum of # verbs, # adjectives, # adverbs, #prepositions, # conjunctions divided by # words |  |  |
|  | **Deictic terms** | # generic words (i.e.: “there”, “here”, “that”); it suggests a tendency to point at, rather than verbally label, items and/or events |  |  |
|  | **Spatial deixis** | # of words referring to place, and spatial elements of the context, mainly through the use of demonstratives |  |  |
|  | **Person deixis** | # of words referring to personal properties of the context, mainly through the use of personal pronouns |  |  |
|  | **Honoré’s statistic** | *NV−0.16;* ; *N* = # word tokens, *V* = # word types |  |  |
|  | **Brunét’s index** | 100log*N*/(1−*V1*/*V*); N = # word tokens, *V1*  = # words used once in the context, *V* = #word types |  |  |
|  | **Type-token ratio** | *#* word types divided by # word tokens | - unique word |  |
|  | **Frequency** | Frequency with which a word occurs in some corpus of natural language; it can be measured for each part of speech (i.e. verb frequency, noun frequency, etc.) |  |  |
|  | **Mean log frequency of nouns** | Mean logarithmic frequency, in a corpus of natural language , of nouns |  |  |
|  | **Familiarity** | Subjective rating of how familiar a word seems |  |  |
|  | **Age of acquisition** | Subjective rating of how old a person is when they first learn that word |  |  |
|  | **Word length** | Mean length, in letters, of each word in the sample |  |  |
|  | **Open- and closed-class word errors** | # errors (subdivided into omission, insertion, and substitution) divided by # open- (or closed-) class words |  |  |
|  | **Semantic errors** | # errors occurring when a target word is replaced by a term that could, from the context, be identified as a semantically related item; this feature includes: semantic (semantically erroneous substitutions) and visual paraphasias (substitutions that are visually similar to the target object) |  |  |
|  | **Word-finding difficulties** (lexical errors) | The proportion of speech comprising word-finding difficulties as indicated by a pause, an immediate repetition of a previous word or production of an indefinite term | Cluster of features: pauses, repetitions, indefinite terms |  |
|  | **Indefinite terms**  (lexical errors) | # empty words without specific meaning; nonspecific nouns or pronouns (i.e., "whatever," "something," "stuff") that made ambiguous or general reference |  |  |
|  | **Revision**  (lexical errors) | The count of pause positions where the speaker retraces a preceding error and then make a correction | - error monitoring | -impaired lexico-semantic domain,  -discourse planning deficits |
|  | **Repetitions**  (lexical errors) | # immediate word repetitions |  | -impaired lexico-semantic domain,  -discourse planning deficits |
|  | **Perseverations** | # items appearing out of context after or before its appearance at the grammatically correct place |  | -impaired lexico-semantic domain,  -discourse planning deficits |
|  | **Repaired sequences** | Sequences of one or more complete words, resulting redundant by subsequent repetitions, elaborations or alternative expressions |  | -impaired lexico-semantic domain,  -syntactic difficulties,  -discourse planning deficits |
|  | **Response to word finding delays** | The most common response to word finding delays, that is whether patients appear unaware of their problem, produce an approximation of the target word or actively search and produce the target word |  | -impaired lexico-semantic domain,  -discourse planning deficits |
|  | **Not in dictionary (NID)**  (lexical errors) | # not in dictionary expressions (i.e.: paraphasias, neologisms) |  | -impaired lexico-semantic domain,  - morpho-syntactic difficulties |
|  | **Circumlocutory comments** | Comment/clauses. Word retrieval difficulty is circumvented by providing a kind of definition of the intended word or phrase. Only identifiable in context of the sentence. |  | -impaired lexico-semantic domain,  -discourse planning deficits |
| Morphosyntactic features | | | | |
|  | **Inflected verbs** | # inflected verbs divided by # verbs |  |  |
|  | **Subject-verb agreement** | Correct agreement of subject and verb |  |  |
|  | **Auxiliary verbs** | # auxiliary verbs divided by # verbs |  |  |
|  | **Gerunds** | # gerund verbs divided by # verbs |  |  |
|  | **Participles** | # participles verbs divided by # verbs |  |  |
|  | **Light verbs** | Number of occurrences of be, have, come, go, give, take, make, do, get, move, and put, normalized by total number of verb |  |  |
|  | **Object clitics** | # object clitics in the speech sample |  |  |
|  | **Grammatical form** | Presence of an appropriate use of syntactic conjunctions, tenses, conditionals, subordinate clauses and passive constructions | - appropriate conjunction | - morpho-syntactic difficulties,  -discourse planning deficits |
|  | **Inflectional errors** | # errors in conjugation and declination of words | - verb agreement errors  - grammatical endings. |  |
|  | **Correct verb tense** | Correct use of verb tense |  | -discourse planning deficits |
| Syntactic features | | | | |
|  | **Clauses** | # clauses ( a clause is a structure consisting of at least a subject and a finite verb) |  |  |
|  | **T-units** | # T-units (a T-units is a clause and all of its dependent clauses). This feature may be also used to calculate: (i) # **T-units per sentences, (ii) # clauses (or sentences) per T-units** |  |  |
|  | **Utterances** | # utterances (a utterance represents any speech sequence consisting of one or more words and preceded and followed by silence) |  |  |
|  | **Mean length of utterance (MLU)** | The average number of morphemes or words per utterance;  this measure can be applied also to:  **mean length of utterance T-units (MLT), mean length of utterance sentences (MLS) or mean length of utterance clauses (MLC)** |  |  |
|  | **Clauses per sentences** | The average number of clauses per sentence; a sentence is a grammatically complete string of words expressing a complete thought, a group of words that forms an independent grammatical unit |  |  |
|  | **Syntax principal component** | Principal component analysis of two features: words in sentences and syntactic errors |  |  |
|  | **Incomplete sentences** | # sentences that are abandoned after producing subjects and verbs | - abandoned sentences  - elliptical phrases | -impaired lexico-semantic domain,  -syntactic difficulties,  -discourse planning deficits |
|  | **Reduced sentences** | # subordinated sentences with nominal verb forms (which are either participles or gerund). To obtain the count for this feature, the frequencies of part of speech tags (gerunds and past participle) are used. |  |  |
|  | **Well-formed sentences** | Percentage of utterances that are well- formed sentences, i.e.: complete sentences free of grammatical errors |  |  |
|  | **Nouns with determiner** | The proportion of nouns with determiner; this feature can be also calculated for compound nouns |  |  |
|  | **Complex nominal** | Noun phrase, clause, or gerund standing for a noun |  |  |
|  | **Noun phrases** | # phrases based on a noun, pronoun, or other noun-like word (nominal) |  |  |
|  | **Verb phrases** | # phrases consisting of at least a verb and its dependents; this feature can be measured on total number of sentences, T-units, etc. | - number of predicates  - Gerund + prepositional phrase constructions |  |
|  | **Embeddings** | # sentences embedded within other sentences |  |  |
|  | **Passive constructions** | # sentences in which the subject receives the action of the verb |  |  |
|  | **Infinitive phrases** | # phrases with infinitive verbs |  |  |
|  | **Prepositional phrases** | # phrases consisting of a preposition, its object(a noun or a pronoun) and any modifiers of the object |  |  |
|  | **Coordinate phrases** | # phrases united by a coordinating conjunction such as “and” | - coordinate phrases per clauses  - coordinate verb phrases |  |
|  | **Dependent clauses** | # clauses that does not form a sentence on its own | - complex units  - subordinate clauses |  |
|  | **Empty phrases** | # utterances with little or no content (i.e.: “and so on”, “something like that”) |  |  |
|  | **Max depth** | Maximum Yngve depth of each parse tree, averaged over all sentences; Yngve depth of a sentence is the maximum number of items in the phrase structure tree that have to be stored during the construction of the sentence |  |  |
|  | **Mean depth** | Mean Yngve depth of each node in the parse tree, averaged over all sentences |  |  |
|  | **Total depth** | Total sum of the Yngve depths of each node in the parse  tree, averaged over all sentences |  |  |
|  | **Tree height** | Height of each parse tree, averaged over all sentences; the height of a tree is the number of edges on the longest downward path between the root and a leaf |  |  |
|  | **Syntactic errors** | # erroneous uses of grammatical rules involving sentence structure or # ungrammatical sentences |  |  |
| Discourse and pragmatic features | | | | |
|  | **Total words** | # produced words |  |  |
|  | Discourse markers | # words or phrases that function primarily as a structuring unit of spoken language (i.e.: “you know”, “you see”, “well”) | Go-ahead utterances |  |
|  | **Cohesion** | # utterances containing:  - referential cohesion (correct pronominal reference)  - temporal cohesion (correct use of verb tense)  - causal cohesion (conjunctions).  Cohesive ties conjoin these elements in the form of references, substitutions, ellipsis, conjunctions, and lexical markers |  |  |
|  | **Correct pronoun** | Correct pronominal reference | - pronouns without antecedent  - referential cohesion  - pronoun substitution/omission  - referent errors |  |
|  | **Local coherence** | The linkage of each event with the preceding event, which is accomplished by rhetorical markers such as sequencing adverbials, pronominal reference to preceding nouns, and statements of cause and effect | - local connectedness |  |
|  | **Global coherence** | A variable that registers whether the speaker acknowledges the point of the story | - global connectedness |  |
|  | **Microproposition** (coherence)  Story narration | # utterances which provide details given in addition to the central topic |  |  |
|  | **Implausible or irrelevant details** | # utterances which provide implausible or irrelevant information given in addition to the central topic | - irrelevant microproposition |  |
|  | **Index of discourse effectiveness** | The ratio of the total number of recalled words divided by the number of macropropositions or information contents |  |  |
|  | **Incomplete content elements** | # utterances conveying part of the essential content of the event or containing missing content elements |  |  |
|  | **Errors in content elements** | # utterances containing factually inaccurate elements |  |  |
|  | **Information content** | # relevant, truthful, non-redundant utterances, excluding phrases containing indefinite terms and redundant words, inappropriate phrases, modalizations and implausible details | - macroproposition  - elaborations  - correct information units |  |
|  | **Ratio words/information** | # words divided by # information contents |  |  |
|  | **Topic maintenance**  (coherence) | Maintenance of the topic; # information content divided by # disruptive topic shifts | - search theme (Frog story) |  |
|  | **Information unit**  (picture description)  or  accurate report  (story narration) | # information units; information units are usually subdivided in subjects, places, objects, and actions (picture description) or in narrative sequences (story narration) | - pictorial theme  - thematic elements  - semantic content  - amount of information  - content unit  - crucial information |  |
|  | **Subjects**  (picture description) | # subjects in the picture |  |  |
|  | **Objects**  (picture description) | # objects in the picture |  |  |
|  | **Places**  (picture description) | # places in the picture |  |  |
|  | **Actions**  (picture description) | # actions in the picture |  |  |
|  | **Efficiency**  (picture description) | # information units divided by duration of speech sample (in seconds) |  |  |
|  | **Ratio words/information unit** | # words divided by # information units |  |  |
|  | **Modalizations** | # speaker’s comments concerning the content of the description including doubts or concerns about their production. |  |  |
|  | **Self-referential tags** | # monitoring comments, i.e.,“don’t I?”, “aren’t I?”, “won’t I?”, etc. |  |  |
|  | **Paraphrasing** | The act of taking what a speaker has been previously stated and reusing it in one’s own context. Three different types of paraphrasing exist: .  - **direct paraphrase** refers to the use of the same word stated by a previous speaker;  - **reflexive form** of paraphrasing refers to cases for which a speaker inverts the pronoun already used by a previous speaker (i.e.: to use “I” when the previous speaker used “you”);  - **indirect paraphrase** of a word occurs when a speaker uses a term related to a previous word stated by another speaker (i.e.: if a first speaker uses the word “farm” and the second speaker uses the word “grow”) |  |  |
|  | **Spontaneity** | # questions or encouraging comments that the interviewer have to insert in order to elicit speech |  |  |

Supplementary Table 2. Results of studies assessing distinct linguistic variables through connected speech in Primary Progressive Aphasia. The results are expressed by reporting the ratio between the studies reporting significant between-group differences and the number of studies inquiring each specific feature. Dark gray rows represent variables significant in at least more than half of the studies considered for each variable (with at least three studies investigating the same feature) or in two out of two studies; the light grey rows represent the features that were tested and found significant in only one study. White rows represent variables not significant. An asterisk (*) is used to highlight the specific studies founding significant between-group differences.

L/Pv = Logopenic variant;

HC = Healthy Controls;

NF/Av = non-fluent variant;

Sv = semantic variant.

| **LINGUISTIC LEVEL** | **FEATURE** | **RESULTS** | **PICTURE DESCRIPTION**  **Ratio References** | | **STORY NARRATION**  **Ratio References** | | **INTERVIEW**  **Ratio References** | | **TOT**  **RATIO** |
| --- | --- | --- | --- | --- | --- | --- | --- | --- | --- |
| **PHONETIC** | Mean first autocorrelation function | NF/Av < Sv | 0/0 | - | 1/1 | Fraser et al., 2013* | 0/0 | - | 1/1 |
|  | Skewness(MFCC) | NF/Av = Sv | 0/0 | - | 0/1 | Fraser et al., 2013 | 0/0 | - | 0/1 |
|  | Kurtosis(MFCC) | NF/Av = Sv | 0/0 | - | 0/1 | Fraser et al., 2013 | 0/0 | - | 0/1 |
|  | Total pause time | NF/Av > HC | 0/0 | - | 1/1 | Ash and Grossman, 2015* | 0/0 | - | 1/1 |
|  |  | Sv > HC | 0/0 | - | 1/1 | Ash and Grossman, 2015* | 0/0 | - | 1/1 |
|  | Mean pauses duration | Sv < HC | 0/0 | - | 0/0 | - | 1/1 | Jarrold et al., 2014* | 1/1 |
|  |  | NF/Av = Sv | 0/0 | - | 0/1 | Fraser et al., 2013 | 0/0 | - | 0/1 |
|  | **Filled pauses** | NF/av > HC | 1/2 | Ash et al., 2013; Wilson et al., 2010* | 1/2 | Fraser et al., 2014a; Fraser et al., 2014b* | 0/0 | - | 2/4 |
|  |  | Sv = HC | 0/1 | Ash et al., 2013 | 0/1 | Fraser et al. 2014a | 0/0 | - | 0/2 |
|  |  | L/Pv > HC | 2/2 | Ash et al., 2013*; Wilson et al., 2010* | 0/0 | - | 0/0 | - | **2/2** |
|  |  | NF/av > Sv | 1/1 | Wilson et al., 2010* | 0/0 | - | 0/0 |  | 1/1 |
|  |  | NF/av = L/Pv | 0/1 | Wilson et al., 2010 | 0/0 | - | 0/0 | - | 0/1 |
|  |  | Sv < L/Pv | 1/1 | Wilson et al., 2010* | 0/0 | - | 0/0 | - | 1/1 |
|  | **Hesitation ratio** | NF/av > HC | 1/1 | Sajjadi et al., 2012b* | 0/0 | - | 1/1 | Sajjadi et al., 2012b* | **2/2** |
|  | Vowel and consonant duration | NF/av > HC | 0/0 | - | 0/0 | - | 1/1 | Jarrold et al., 2014* | 1/1 |
|  | **Speech rate** | NF/av < HC | 4/4 | Ash et al., 2013*; Graham et al., 2004*; Wilson et al., 2010*; Sajjadi et al., 2012b* | 3/3 | Ash and Grossman, 2015*; Ash et al., 2006*; Fraser et al., 2014a* | 2/2 | Knibb et al., 2009*; Sajjadi et al., 2012b* | **9/9** |
|  |  | Sv < HC | 3/4 | Ash et al., 2013*; Wilson et al., 2010*; Sajjadi et al., 2012a*; Garrard and Forsyth, 2010 | 3/3 | Ash and Grossman, 2015*; Ash et al., 2006*; Fraser et al., 2014a* | 1/1 | Sajjadi et al., 2012a* | **7/8** |
|  |  | L/Pv < HC | 2/2 | Ash et al., 2013*; Wilson et al., 2010* | 1/1 | Ash and Grossman, 2015*; | 0/0 | - | **3/3** |
|  |  | NF/av < Sv | 2/2 | Ash et al., 2013*; Wilson et al., 2010* | 1/2 | Ash et al. 2006*; Fraser et al., 2014a | 0/0 | - | **3/4** |
|  |  | NF/av < L/Pv | 1/2 | Ash et al., 2013*; Wilson et al., 2010 | 0/0 | - | 0/0 | - | 1/2 |
|  |  | Sv > L/Pv | 2/2 | Ash et al., 2013*; Wilson et al., 2010* | 0/0 | - | 0/0 | - | **2/2** |
|  | Maximum speech rate | NF/av < HC | 1/1 | Wilson et al., 2010* | 0/0 | - | 0/0 | - | 1/1 |
|  |  | Sv = HC | 0/1 | Wilson et al., 2010 | 0/0 | - | 0/0 | - | 0/1 |
|  |  | L/Pv < HC | 1/1 | Wilson et al.,, 2010* | 0/0 | - | 0/0 | - | 1/1 |
|  |  | NF/av < Sv | 1/1 | Wilson et al., 2010* | 0/0 | - | 0/0 | - | 1/1 |
|  |  | NF/av < L/Pv | 1/1 | Wilson et al., 2010* | 0/0 | - | 0/0 | - | 1/1 |
|  |  | Sv > L/Pv | 1/1 | Wilson et al., 2010* | 0/0 | - | 0/0 | - | 1/1 |
|  | Total phonation time | NF/av = Sv |  |  | 0/1 | Fraser et al., 2013 | 0/0 | - | 0/1 |
|  | **Total locution time** | NF/av > HC | 2/2 | Graham et al., 2004* Wilson et al., 2010* | 1/1 | Ash and Grossman, 2015* | 0/0 | - | **3/3** |
|  |  | Sv = HC | 0/1 | Wilson et al., 2010 | 0/1 | Ash and Grossman, 2015 | 0/0 | - | 0/2 |
|  |  | L/Pv = HC | 0/1 | Wilson et al., 2010 | 0/0 | - | 0/0 | - | 0/1 |
|  |  | NF/av = Sv | 0/1 | Wilson et al., 2010 | 0/0 | - | 0/0 | - | 0/1 |
|  |  | NF/av = L/Pv | 0/1 | Wilson et al., 2010 | 0/0 | - | 0/0 | - | 0/1 |
|  |  | Sv = L/Pv | 0/1 | Wilson et al., 2010 | 0/0 | - | 0/0 | - | 0/1 |
|  | Phonetic errors | NF/av > HC | 1/2 | Wilson et al., 2010*; Ash et al., 2013 | 0/0 | - | 0/0 | - | 1/2 |
|  |  | Sv =HC | 0/1 | Ash et al., 2013 | 0/0 | - | 0/0 | - | 0/1 |
|  |  | L/Pv = HC | 0/1 | Ash et al., 2013 | 0/0 | - | 0/0 | - | 0/1 |
|  |  | NF/av > Sv | 1/2 | Wilson et al., 2010*; Ash et al., 2013 | 0/0 | - | 0/0 | - | 1/2 |
|  |  | NF/av > L/Pv | 1/2 | Wilson et al., 2010*; Ash et al., 2013 | 0/0 | - | 0/0 | - | 1/2 |
|  |  | Sv = L/Pv | 0/2 | Wilson et al., 2010; Ash et al., 2013 | 0/0 | - | 0/0 | - | 0/2 |
| **PHONOLOGICAL** | **Phonemic errors** | NF/av > HC | 3/4 | Ash et al., 2013*; Graham et al., 2004*; Sajjadi et al., 2012b*; Wilson et al., 2010 | 1/1 | Ash and Grossman, 2015* | 2/2 | Knibb et al., 2009*; Sajjadi et al., 2012b* | **6/7** |
|  |  | Sv > HC | 0/2 | Ash et al., 2013; Sajjadi et al., 2012a | 1/1 | Ash and Grossman, 2015* | 0/1 | Sajjadi et al., 2012a | 1/4 |
|  |  | L/Pv > HC | 1/2 | Ash et al., 2013; Wilson et al., 2010* | 1/1 | Ash and Grossman, 2015* | 0/0 | - | **2/3** |
|  |  | NF/av = Sv | 0/2 | Ash et al., 2013; Wilson et al., 2010 | 0/0 | - | 0/0 | - | 0/2 |
|  |  | NF/av = L/Pv | 0/2 | Ash et al., 2013; Wilson et al., 2010 | 0/0 | - | 0/0 | - | 0/2 |
|  |  | Sv = L/Pv | 0/2 | Ash et al., 2013; Wilson et al., 2010 | 0/0 | - | 0/0 | - | 0/2 |
|  | **False starts** | NF/av > HC | 1/2 | Sajjadi et al., 2012b*; Wilson et al., 2010 | 0/0 | - | 1/1 | Sajjadi et al., 2012b* | **2/3** |
|  |  | Sv > HC | 1/1 | Sajjadi et al., 2012a* | 0/0 | - | 2/2 | Meteyard and Patterson, 2009*; Sajjadi et al., 2012a* | **3/3** |
|  |  | L/Pv > HC | 1/1 | Wilson et al., 2010* | 0/0 | - | 0/0 | - | 1/1 |
|  |  | NF/av = Sv | 0/1 | Wilson et al., 2010 | 0/0 | - | 0/0 | - | 0/1 |
|  |  | NF/av = L/Pv | 0/1 | Wilson et al., 2010 | 0/0 | - | 0/0 | - | 0/1 |
|  |  | Sv = L/Pv | 0/1 | Wilson et al., 2010 | 0/0 | - | 0/0 | - | 0/1 |
| **LEXICO-SEMANTIC** | **Noun rate** | NF/av =HC | 0/2 | Ash et al., 2013; Graham et al., 2004 | 0/2 | Ash and Grossman, 2015; Fraser et al., 2014a | 0/0 | - | 0/4 |
|  |  | Sv < HC | 1/1 | Ash et al., 2013* | 2/2 | Ash and Grossman, 2015*; Fraser et al., 2014a* | 1/1 | Jarrold et al., 2014* | **4/4** |
|  |  | L/Pv < HC | 0/1 | Ash et al., 2013 | 1/1 | Ash and Grossman, 2015* | 0/0 | - | 1/2 |
|  |  | NF/av > Sv | 1/1 | Ash et al., 2013* | 0/1 | Fraser et al., 2014a | 0/0 | - | 1/2 |
|  |  | NF/av > L/Pv | 1/1 | Ash et al., 2013* | 0/0 | - | 0/0 | - | 1/1 |
|  |  | Sv = L/Pv | 0/1 | Ash et al., 2013 | 0/0 | - | 0/0 | - | 0/1 |
|  | Verb rate | NF/av = HC | 0/2 | Graham et al., 2004; Wilson et al., 2010 | 0/1 | Fraser et al., 2014a, | 0/0 | - | 0/3 |
|  |  | Sv > HC | 1/1 | Wilson et al., 2010* | 0/1 | Fraser et al., 2014a | 0/0 | - | 1/2 |
|  |  | L/Pv = HC | 0/1 | Wilson et al., 2010 | 0/0 | - | 0/0 | - | 0/1 |
|  |  | NF/av < Sv | 1/1 | Wilson et al., 2010* | 0/1 | Fraser et al., 2014a | 0/0 | - | 1/2 |
|  |  | NF/av < L/Pv | 1/1 | Wilson et al., 2010* | 0/0 | - | 0/0 | - | 1/1 |
|  |  | Sv = L/Pv | 0/1 | Wilson et al., 2010 |  |  | 0/0 | - | 0/1 |
|  | Demonstrative rate | NF/av > HC | 0/0 | - | 1/1 | Fraser et al., 2014a* | 0/0 | - | 1/1 |
|  |  | Sv > HC | 0/0 | - | 1/1 | Fraser et al., 2014a* | 0/0 | - | 1/1 |
|  |  | NF/av = Sv | 0/0 | - | 0/1 | Fraser et al., 2014a | 0/0 | - | 0/1 |
|  | Adjective rate | NF/av = HC | 0/0 | - | 0/1 | Fraser et al., 2014a | 0/0 | - | 0/1 |
|  |  | Sv = HC | 0/0 | - | 0/1 | Fraser et al., 2014a | 0/0 | - | 0/1 |
|  |  | NF/av = Sv | 0/0 | - | 0/1 | Fraser et al., 2014a | 0/0 | - | 0/1 |
|  | **Pronoun rate** | NF/av = HC | 0/1 | Wilson et al., 2010 | 0/0 | - | 0/0 | - | 0/1 |
|  |  | Sv > HC | 1/1 | Wilson et al., 2010* | 0/0 | - | 1/1 | Jarrold et al., 2014* | **2/2** |
|  |  | L/Pv > HC | 1/1 | Wilson et al., 2010* | 0/0 | - | 0/0 | - | 1/1 |
|  |  | NF/av < Sv | 1/1 | Wilson et al., 2010* | 0/0 | - | 0/0 | - | 1/1 |
|  |  | NF/av < L/Pv | 1/1 | Wilson et al., 2010* | 0/0 | - | 0/0 | - | 1/1 |
|  |  | Sv > L/Pv | 1/1 | Wilson et al., 2010* | 0/0 | - | 0/0 | - | 1/1 |
|  | Adverb rate | NF/av = HC | 0/0 | - | 0/1 | Fraser et al., 2014a | 0/0 | - | 0/1 |
|  |  | Sv > HC | 0/0 | - | 1/1 | Fraser et al., 2014a* | 0/0 | - | 1/1 |
|  |  | NF/av = Sv | 0/0 | - | 0/1 | Fraser et al., 2014a | 0/0 | - | 0/1 |
|  | Possessive rate | NF/av < HC | 0/0 | - | 1/1 | Fraser et al., 2014a* | 0/0 | - | 1/1 |
|  |  | Sv = HC | 0/0 | - | 0/1 | Fraser et al., 2014a | 0/0 | - | 0/1 |
|  | **Noun-verb ratio** | NF/av = HC | 0/1 | Graham et al., 2004 | 0/1 | Fraser et al., 2014a | 0/1 | Knibb et al., 2009 | 0/3 |
|  |  | Sv < HC | 1/1 | Garrard and Forsyth, 2010* | 1/1 | Fraser et al., 2014a* | 0/0 | - | **2/2** |
|  |  | NF/av = Sv | 0/0 | - | 0/1 | Fraser et al., 2014a | 0/0 | - | 0/1 |
|  | Noun ratio | NF/av = HC | 0/0 | - | 0/1 | Fraser et al., 2014a | 0/0 | - | 0/1 |
|  |  | Sv < HC | 0/0 | - | 1/1 | Fraser et al., 2014a* | 0/0 | - | 1/1 |
|  |  | NF/av = Sv | 0/0 | - | 0/1 | Fraser et al., 2014a | 0/0 | - | 0/1 |
|  | Pronoun ratio | NF/av = HC | 0/0 | - | 0/1 | Fraser et al., 2014a | 0/0 | - | 0/1 |
|  |  | Sv > HC | 0/0 | - | 1/1 | Fraser et al., 2014a* | 0/0 | - | 1/1 |
|  |  | NF/av = Sv | 0/0 | - | 0/1 | Fraser et al., 2014a | 0/0 | - | 0/1 |
|  | Closed-class words | NF/av < HC | 0/3 | Graham et al., 2004, Wilson et al., 2010, Sajjadi et al., 2012b | 0/1 | Fraser et al., 2014a | 1/2 | Jarrold et al., 2014*; Sajjadi et al., 2012b | 1/6 |
|  |  | Sv > HC | 1/1 | Sajjadi et al., 2012a* | 0/1 | Fraser et al., 2014a | 0/1 | Sajjadi et al., 2012a | 1/3 |
|  |  | L/Pv = HC | 0/1 | Wilson et al., 2010 | 0/0 | - | 0/0 | - | 0/1 |
|  |  | NF/av < Sv | 1/1 | Wilson et al., 2010* | 0/1 | Fraser et al., 2014a | 0/0 | - | 1/2 |
|  |  | NF/av < L/Pv | 1/1 | Wilson et al., 2010* | 0/0 | - | 0/0 | - | 1/1 |
|  |  | Sv = L/Pv | 0/1 | Wilson et al., 2010 | 0/0 | - | 0/0 | - | 0/1 |
|  | Open class words | NF/av = HC | 0/2 | Ash et al., 2013, Graham et al., 2004 |  |  | 0/1 | Knibb et al., 2009 | 0/3 |
|  |  | Sv < HC | 1/1 | Ash et al., 2013* | 0/0 | - | 0/0 | - | 1/1 |
|  |  | L/Pv < HC | 1/1 | Ash et al., 2013* | 0/0 | - | 0/0 | - | 1/1 |
|  |  | NF/av = Sv | 0/1 | Wilson et al., 2010 | 0/0 | - | 0/0 | - | 0/1 |
|  |  | NF/av = L/Pv | 0/1 | Wilson et al., 2010 | 0/0 | - | 0/0 | - | 0/1 |
|  |  | Sv = L/Pv | 0/1 | Wilson et al., 2010 | 0/0 | - | 0/0 | - | 0/1 |
|  | Content Density | Sv < HC | 1/1 | Garrard and Forsyth, 2010* | 0/0 | - | 0/0 | - | 1/1 |
|  | Type-token ratio | NF/av = HC | 0/0 | - | 0/1 | Fraser et al., 2014a | 0/0 | - | 0/1 |
|  |  | Sv = HC | 0/1 | Garrard and Forsyth, 2010 | 0/1 | Fraser et al., 2014a | 0/0 | - | 0/2 |
|  |  | NF/av = Sv | 0/0 | - | 0/1 | Fraser et al., 2014a | 0/0 | - | 0/1 |
|  | Frequency | NF/av > HC | 0/0 | - | 1/1 | Fraser et al., 2014a* | 0/1 | Jarrold et al., 2014 | 1/2 |
|  |  | Sv > HC | 0/0 | - | 1/1 | Fraser et al., 2014a* | 0/0 | - | 1/1 |
|  | Noun frequency | Sv > HC | 0/0 | - | 1/1 | Fraser et al., 2014a* | 0/0 | - | 1/1 |
|  |  | NF/av < Sv | 0/0 | - | 1/2 | Fraser et al., 2014a*; Fraser et al., 2013 | 0/0 | - | 1/2 |
|  | Verb frequency | NF/av > HC | 0/0 | - | 1/1 | Fraser et al., 2014a* | 0/0 | - | 1/1 |
|  | Mean log frequency of nouns | NF/av = HC | 0/1 | Wilson et al., 2010 | 0/0 | - | 0/0 | - | 0/1 |
|  |  | Sv > HC | 1/1 | Wilson et al., 2010* | 0/0 | - | 0/0 | - | 1/1 |
|  |  | L/Pv = HC | 0/1 | Wilson et al., 2010 | 0/0 | - | 0/0 | - | 0/1 |
|  |  | NF/av < Sv | 1/1 | Wilson et al., 2010* | 0/0 | - | 0/0 | - | 1/1 |
|  |  | NF/av = L/Pv | 0/1 | Wilson et al., 2010 | 0/0 | - | 0/0 | - | 0/1 |
|  |  | Sv > L/Pv | 1/1 | Wilson et al., 2010* | 0/0 | - | 0/0 | - | 1/1 |
|  | Familiarity | NF/av = HC | 0/0 | - | 0/1 | Fraser et al., 2014a | 0/0 | - | 0/1 |
|  |  | NF/av < Sv | 0/0 | - | 1/1 | Fraser et al., 2013* | 0/0 | - | 1/1 |
|  | **Noun familiarity** | Sv > HC | 0/0 | - | 1/1 | Fraser et al., 2014a* | 0/0 | - | 1/1 |
|  |  | NF/av < Sv | 0/0 | - | 2/2 | Fraser et al., 2013*; Fraser et al., 2014a* | 0/0 | - | **2/2** |
|  | Age of acquisition | NF/av < HC | 0/0 | - | 1/1 | Fraser et al., 2014a* | 0/0 | - | 1/1 |
|  |  | Sv = HC | 0/0 | - | 0/1 | Fraser et al., 2014a | 0/0 | - | 0/1 |
|  |  | NF/av = Sv | 0/0 | - | 0/1 | Fraser et al., 2014a | 0/0 | - | 0/1 |
|  | Noun age of acquisition | NF/av < HC | 0/0 | - | 1/1 | Fraser et al., 2014a* | 0/0 | - | 1/1 |
|  | Imageability | NF/av = HC | 0/0 | - | 0/1 | Fraser et al., 2014a | 0/0 | - | 0/1 |
|  |  | NF/av = Sv | 0/0 | - | 0/2 | Fraser et al., 2013, | 0/0 | - | 0/2 |
|  | Verb imageability | Sv > HC | 0/0 | - | 1/1 | Fraser et al., 2014a* | 0/0 | - | 1/1 |
|  | Word length | NF/av < HC | 0/0 | - | 1/1 | Fraser et al., 2014a* | 0/0 | - | 1/1 |
|  |  | Sv < HC | 0/0 | - | 1/1 | Fraser et al., 2014a* | 0/0 | - | 1/1 |
|  |  | NF/av = Sv | 0/0 | - | 0/2 | Fraser et al., 2013; Fraser et al., 2014a | 0/0 | - | 0/2 |
|  | Open class word errors | NF/av > HC | 1/1 | Sajjadi et al., 2012b* | 0/0 | - | 0/1 | Sajjadi et al., 2012b | 1/2 |
|  |  | Sv > HC | 0/1 | Sajjadi et al., 2012a | 0/0 | - | 1/2 | Meteyard and Patterson, 2009*; Sajjadi et al., 2012a | 1/3 |
|  | **Closed class word errors** | NF/av > HC | 0/1 | Sajjadi et al., 2012b | 0/0 | - | 2/2 | Knibb et al., 2009*; Sajjadi et al., 2012b* | **2/3** |
|  |  | Sv > HC | 0/1 | Sajjadi et al., 2012a | 0/0 | - | 1/2 | Meteyard and Patterson, 2009*; Sajjadi et al., 2012a | 1/3 |
|  | **Semantic errors** | NF/av > HC | 0/2 | Wilson et al., 2010; Sajjadi et al., 2012b | 0/0 | - | 1/2 | Knibb et al., 2009*; Sajjadi et al., 2012b | 1/4 |
|  |  | Sv > HC | 1/1 | Sajjadi et al., 2012a* | 0/0 | - | 2/2 | Meteyard and Patterson, 2009*; Sajjadi et al., 2012a* | **3/3** |
|  |  | L/Pv = HC | 0/1 | Wilson et al., 2010 | 0/0 | - | 0/0 | - | 0/1 |
|  |  | NF/av = Sv | 0/1 | Wilson et al., 2010 | 0/0 | - | 0/0 | - | 0/1 |
|  |  | NF/av = L/Pv | 0/1 | Wilson et al., 2010 | 0/0 | - | 0/0 | - | 0/1 |
|  |  | Sv = L/Pv | 0/1 | Wilson et al., 2010 | 0/0 | - | 0/0 | - | 0/1 |
|  | Word-finding difficulties (lexical errors) | NF/av = HC | 0/0 | - | 0/1 | Ash et al., 2006 | 0/0 | - | 0/1 |
|  |  | Sv > HC | 0/0 | - | 1/1 | Ash et al., 2006* | 0/0 | - | 1/1 |
|  |  | NF/av = Sv | 0/0 | - | 0/1 | Ash et al., 2006 | 0/0 | - | 0/1 |
|  | Indefinite terms  (lexical errors) | Sv > HC | 0/0 | - | 0/0 | - | 1/1 | Meteyard and Patterson, 2009* | 1/1 |
|  | Revision  (lexical errors) | Sv = HC | 0/0 | - | 0/0 | - | 0/1 | Meteyard and Patterson, 2009 | 0/1 |
|  | Perseveration | Sv > HC | 0/0 | - | 0/0 | - | 1/1 | Meteyard and Patterson, 2009* | 1/1 |
|  | **Repaired sequences** | NF/av > HC | 0/1 | Wilson et al., 2010 | 1/1 | Ash and Grossman, 2015* | 0/0 | - | 1/2 |
|  |  | Sv > HC | 1/2 | Sajjadi et al., 2012a*; Wilson et al., 2010 | 0/1 | Ash and Grossman, 2015 | 1/1 | Sajjadi et al., 2012a* | 2/4 |
|  |  | L/Pv > HC | 1/1 | Wilson et al., 2010* | 1/1 | Ash and Grossman, 2015* |  |  | **2/2** |
|  |  | NF/av = Sv | 0/1 | Wilson et al., 2010 | 0/0 | - | 0/0 | - | 0/1 |
|  |  | NF/av = L/Pv | 0/1 | Wilson et al., 2010 | 0/0 | - | 0/0 | - | 0/1 |
|  |  | Sv < L/Pv | 1/1 | Wilson et al., 2010* | 0/0 | - | 0/0 | - | 1/1 |
|  | Not in dictionary (lexical errors) | NF/av > HC | 1/1 | Graham et al., 2004* | 0/0 | - | 0/0 | - | 1/1 |
|  | **Circumlocutory comments** | Sv > HC | 1/1 | Sajjadi et al., 2012a* | 0/0 | - | 1/1 | Sajjadi et al., 2012a* | **2/2** |
| **MORPHOSYNTACTIC** | Inflected verbs | NF/av = HC | 0/1 | Wilson et al., 2010 | 0/2 | Ash and Grossman, 2015; Fraser et al., 2014a | 0/1 | Knibb et al., 2009 | 0/4 |
|  |  | Sv < HC | 0/1 | Wilson et al., 2010 | 0/2 | Ash and Grossman, 2015; Fraser et al., 2014a | 1/1 | Meteyard and Patterson, 2009* | 1/4 |
|  |  | L/Pv = HC | 0/1 | Wilson et al., 2010 | 0/1 | Ash and Grossman, 2015 | 0/0 | - | 0/2 |
|  |  | NF/av = Sv | 0/1 | Wilson et al., 2010 | 0/1 | Fraser et al., 2014a | 0/0 | - | 0/2 |
|  |  | NF/av = L/Pv | 0/1 | Wilson et al., 2010 | 0/0 | - | 0/0 | - | 0/1 |
|  |  | Sv = L/Pv | 0/1 | Wilson et al., 2010 | 0/0 | - | 0/0 | - | 0/1 |
|  | Subject-verb agreement | Sv < HC | 0/1 | Sajjadi et al., 2012a | 0/0 | - | 1/2 | Sajjadi et al., 2012a*; Meteyard and Patterson, 2009 | 1/3 |
|  | Auxiliary verbs | NF/av = HC | 0/1 | Wilson et al., 2010 | 0/0 | - | 0/0 | - | 0/1 |
|  |  | NF/av = Sv | 0/1 | Wilson et al., 2010 | 0/0 | - | 0/0 | - | 0/1 |
|  |  | NF/av = L/Pv | 0/1 | Wilson et al., 2010 | 0/0 | - | 0/0 | - | 0/1 |
|  |  | Sv = L/Pv | 0/1 | Wilson et al., 2010 | 0/0 | - | 0/0 | - | 0/1 |
|  | Light verbs | NF/av = HC | 0/0 | - | 0/1 | Fraser et al., 2014a | 0/0 | - | 0/1 |
|  |  | Sv > HC | 0/0 | - | 0/1 | Fraser et al., 2014a | 1/1 | Meteyard and Patterson, 2009* | 1/2 |
|  |  | NF/av = Sv | 0/0 | - | 0/1 | Fraser et al., 2014a | 0/0 | - | 0/1 |
|  | Grammatical form | NF/av = HC | 0/1 | Graham et al., 2004 | 0/0 | - | 0/0 | - | 0/1 |
|  | **Inflectional errors** | NF/av > HC | 2/2 | Graham et al., 2004*; Sajjadi et al., 2012b* | 0/0 | - | 1/1 | Sajjadi et al., 2012b* | **3/3** |
|  |  | Sv > HC | 1/1 | Sajjadi et al., 2012a* | 0/0 | - | 1/1 | Sajjadi et al., 2012a* | **2/2** |
| **SYNTACTIC** | Clauses | NF/av < HC | 0/0 | - | 1/2 | Fraser et al., 2015b*; Fraser et al., 2014a | 0/0 | - | 1/2 |
|  |  | Sv > HC | 0/0 | - | 1/2 | Fraser et al., 2015b, Fraser et al., 2014a* | 0/0 | - | 1/2 |
|  |  | NF/av = Sv | 0/0 | - | 0/1 | Fraser et al., 2014a | 0/0 | - | 0/1 |
|  | T-units | NF/av < HC | 0/0 | - | 1/2 | Fraser et al., 2015b*; Fraser et al., 2014a | 0/0 | - | 1/2 |
|  |  | Sv = HC | 0/0 | - | 0/2 | Fraser et al., 2015b; Fraser et al., 2014a | 0/0 | - | 0/2 |
|  |  | NF/av = Sv | 0/0 | - | 0/1 | Fraser et al., 2014a | 0/0 | - | 0/1 |
|  | Sentences | NF/av = HC | 0/0 | - | 0/2 | Fraser et al., 2015b; Fraser et al., 2014a | 0/0 | - | 0/2 |
|  |  | Sv = HC | 0/0 | - | 0/2 | Fraser et al., 2015b; Fraser et al., 2014a | 0/0 | - | 0/2 |
|  |  | NF/av = Sv | 0/0 | - | 0/1 | Fraser et al., 2014a | 0/0 | - | 0/1 |
|  | Utterances | NF/av = HC | 0/0 | - | 0/1 | Ash and Grossman, 2015 | 0/0 | - | 0/1 |
|  |  | Sv = HC | 0/0 | - | 0/1 | Ash and Grossman, 2015 | 0/0 | - | 0/1 |
|  |  | L/Pv = HC | 0/0 | - | 0/1 | Ash and Grossman, 2015 | 0/0 | - | 0/1 |
|  | **Mean length of utterance** | NF/av < HC | 3/3 | Ash et al., 2013*; Wilson et al., 2010*; Sajjadi et al., 2012b* | 2/3 | Ash and Grossman, 2015*; Ash et al., 2006*; Fraser et al., 2014a | 1/1 | Sajjadi et al., 2012b* | **6/7** |
|  |  | Sv < HC | 1/2 | Ash et al., 2013, Wilson et al., 2010*; | 1/2 | Ash and Grossman, 2015*; Ash et al., 2006 | 1/1 | Meteyard and Patterson, 2009*; | **3/5** |
|  |  | L/Pv < HC | 1/2 | Ash et al., 2013, Wilson et al., 2010* | 0/1 | Ash and Grossman, 2015 | 0/0 | - | 1/3 |
|  |  | NF/av < Sv | 1/2 | Wilson et al., 2010*; Ash et al., 2013 | 0/2 | Ash et al., 2006; Fraser et al., 2014a | 0/0 | - | 1/4 |
|  |  | NF/av < L/Pv | 1/2 | Ash et al., 2013*; Wilson et al., 2010 | 0/0 | - | 0/0 | - | 1/2 |
|  |  | Sv = L/Pv | 0/2 | Ash et al., 2013, Wilson et al., 2010 | 0/0 | - | 0/0 | - | 0/2 |
|  | Mean length of clause | NF/av < HC | 0/0 | - | 1/1 | Fraser et al., 2015b* | 0/0 | - | 1/1 |
|  |  | Sv < HC | 0/0 | - | 1/2 | Fraser et al., 2015b*; Fraser et al., 2014a | 0/0 | - | 1/2 |
|  | **Mean length of T-unit** | NF/av < HC | 0/0 | - | 1/1 | Fraser et al., 2015b* | 0/0 | - | 1/1 |
|  |  | Sv < HC | 1/1 | Sajjadi et al., 2012a* | 1/1 | Fraser et al., 2015b* | 1/1 | Sajjadi et al., 2012a* | **3/3** |
|  | **Mean length of sentence** | NF/av < HC | 0/0 | - | 1/1 | Fraser et al., 2015b* | 1/1 | Knibb et al., 2009* | **2/2** |
|  |  | Sv < HC | 0/0 | - | 1/2 | Fraser et al., 2015b*; Fraser et al., 2014a | 0/0 | - | 1/2 |
|  | Clauses per sentences | NF/av < HC | 0/0 | - | 1/1 | Fraser et al., 2015b* | 0/0 | - | 1/1 |
|  |  | Sv < HC | 0/0 | - | 1/2 | Fraser et al., 2015b*; Fraser et al., 2014a | 0/0 | - | 1/2 |
|  |  | NF/av > Sv | 0/0 | - | 1/1 | Fraser et al., 2015b* | 0/0 | - | 1/1 |
|  | Clauses per T-units | Sv < HC | 0/0 | - | 1/1 | Fraser et al., 2015b* | 0/0 | - | 1/1 |
|  | T-unit per sentence | NF/av < HC | 0/0 | - | 1/1 | Fraser et al., 2014a* | 0/0 | - | 1/1 |
|  | Syntax principal component | NF/av < HC | 1/1 | Wilson et al., 2010* | 0/0 | - | 0/0 | - | 1/1 |
|  |  | Sv < HC | 1/1 | Wilson et al., 2010* | 0/0 | - | 0/0 | - | 1/1 |
|  |  | L/Pv < HC | 1/1 | Wilson et al., 2010* | 0/0 | - | 0/0 | - | 1/1 |
|  |  | NF/av = Sv | 0/1 | Wilson et al., 2010 | 0/0 | - | 0/0 | - | 0/1 |
|  |  | NF/av = L/Pv | 0/1 | Wilson et al., 2010 | 0/0 | - | 0/0 | - | 0/1 |
|  |  | Sv > L/Pv | 1/1 | Wilson et al., 2010* | 0/0 | - | 0/0 | - | 1/1 |
|  | **Incomplete sentences** (Fluency disruption) | NF/av > HC | 1/2 | Wilson et al., 2010, Sajjadi et al., 2012b* | 0/0 | - | 2/2 | Knibb et al., 2009*; Sajjadi et al., 2012b* | **3/4** |
|  |  | Sv > HC | 1/1 | Wilson et al., 2010* | 0/0 | - | 0/0 | - | 1/1 |
|  |  | L/Pv = HC | 0/1 | Wilson et al., 2010 | 0/0 | - | 0/0 | - | 0/1 |
|  |  | NF/av < Sv | 1/1 | Wilson et al., 2010* | 0/0 | - | 0/0 | - | 1/1 |
|  |  | NF/av = L/Pv | 0/1 | Wilson et al., 2010 | 0/0 | - | 0/0 | - | 0/1 |
|  |  | Sv = L/Pv | 0/1 | Wilson et al., 2010 | 0/0 | - | 0/0 | - | 0/1 |
|  | **Reduced sentences** | Sv > HC | 1/1 | Sajjadi et al., 2012a* | 0/0 | - | 1/1 | Sajjadi et al., 2012a* | **2/2** |
|  | **Well-formed sentences** | NF/av < HC | 0/1 | Ash et al., 2013 | 1/1 | Ash and Grossman, 2015* | 0/0 | - | 1/2 |
|  |  | Sv < HC | 1/1 | Ash et al., 2013* | 1/1 | Ash and Grossman, 2015* | 0/0 | - | **2/2** |
|  |  | L/Pv < HC | 1/1 | Ash et al., 2013* | 1/1 | Ash and Grossman, 2015* | 0/0 | - | **2/2** |
|  |  | NF/av = Sv | 0/1 | Ash et al., 2013 | 0/0 | - | 0/0 | - | 0/1 |
|  |  | NF/av = L/Pv | 0/1 | Ash et al., 2013 | 0/0 | - | 0/0 | - | 0/1 |
|  |  | Sv = L/Pv | 0/1 | Ash et al., 2013 | 0/0 | - | 0/0 | - | 0/1 |
|  | **Nouns with determiner** | NF/av < HC | 1/1 | Wilson et al., 2010* | 1/1 | Fraser et al., 2014b* | 0/0 | - | **2/2** |
|  |  | Sv = HC | 0/1 | Wilson et al., 2010 | 0/0 | - | 0/0 | - | 0/1 |
|  |  | L/Pv = HC | 0/1 | Wilson et al., 2010 | 0/0 | - | 0/0 | - | 0/1 |
|  |  | NF/av = Sv | 0/1 | Wilson et al., 2010 | 0/0 | - | 0/0 | - | 0/1 |
|  |  | NF/av = L/Pv | 0/1 | Wilson et al., 2010 | 0/0 | - | 0/0 | - | 0/1 |
|  |  | Sv = L/Pv | 0/1 | Wilson et al., 2010 | 0/0 | - | 0/0 | - | 0/1 |
|  | Noun modified by possessive pronoun + adjective | NF/av < HC | 0/0 | - | 1/1 | Fraser et al., 2014b* | 0/0 | - | 1/1 |
|  | Complex nominal | NF/av < HC | 0/0 | - | 1/2 | Fraser et al., 2015b*; Fraser et al., 2014a | 0/0 | - | 1/2 |
|  |  | Sv = HC | 0/0 | - | 0/2 | Fraser et al., 2015b; Fraser et al., 2014a | 0/0 | - | 0/2 |
|  |  | NF/av = Sv | 0/0 | - | 0/1 | Fraser et al., 2014a | 0/0 | - | 0/1 |
|  | Noun phrases | NF/av > HC | 0/0 | - | 1/1 | Fraser et al., 2014b* | 0/0 | - | 1/1 |
|  | **Verb phrases** | NF/av < HC | 0/0 | - | 2/3 | Fraser et al., 2015b*; Fraser et al., 2014b*; Fraser et al., 2014a | 0/0 | - | **2/3** |
|  |  | Sv = HC | 0/0 | - | 0/2 | Fraser et al., 2015b; Fraser et al., 2014a | 0/0 | - | 0/2 |
|  |  | NF/av = Sv | 0/0 | - | 0/1 | Fraser et al., 2014a | 0/0 | - | 0/1 |
|  | Verb phrases per T-units | NF/av < HC | 0/0 | - | 1/1 | Fraser et al., 2015b* | 0/0 | - | 1/1 |
|  |  | Sv < HC | 0/0 | - | 1/1 | Fraser et al., 2015b* | 0/0 | - | 1/1 |
|  | **Embeddings** | NF/av < HC | 0/1 | Wilson et al., 2010 | 1/1 | Fraser et al., 2014b* | 1/1 | Knibb et al., 2009* | **2/3** |
|  |  | Sv > HC | 1/1 | Wilson et al., 2010* | 0/0 | - | 0/0 | - | 1/1 |
|  |  | L/Pv = HC | 0/1 | Wilson et al., 2010 | 0/0 | - | 0/0 | - | 0/1 |
|  |  | NF/av < Sv | 1/1 | Wilson et al., 2010* | 0/0 | - | 0/0 | - | 1/1 |
|  |  | NF/av = L/Pv | 0/1 | Wilson et al., 2010 | 0/0 | - | 0/0 | - | 0/1 |
|  |  | Sv > L/Pv | 1/1 | Wilson et al., 2010* | 0/0 | - | 0/0 | - | 1/1 |
|  | Passive construction | NF/av < HC | 0/0 | - |  |  | 1/1 | Knibb et al., 2009* | 1/1 |
|  | Infinitive phrases | NF/av < HC | 0/0 | - | 1/1 | Fraser et al., 2014b* | 0/0 | - | 1/1 |
|  | Prepositional phrases | NF/av < HC | 0/0 | - | 1/1 | Fraser et al., 2014b* | 0/0 | - | 1/1 |
|  | **Coordinate sentences** | NF/av < HC | 0/0 | - | 2/3 | Fraser et al., 2014b*; Fraser et al., 2015b*; Fraser et al., 2014a | 0/0 | - | **2/3** |
|  |  | Sv < HC | 0/0 | - | 1/2 | Fraser et al., 2015b*; Fraser et al., 2014a | 0/0 | - | 1/2 |
|  |  | NF/av = Sv | 0/0 | - | 0/1 | Fraser et al., 2014a | 0/0 | - | 0/1 |
|  | **Dependent clauses** | NF/av < HC | 2/2 | Ash et al., 2013*; Sajjadi et al., 2012b* | 2/3 | Ash and Grossman, 2015*; Fraser et al., 2015b*; Fraser et al., 2014a | 2/2 | Knibb et al., 2009*; Sajjadi et al., 2012b* | **6/7** |
|  |  | Sv < HC | 1/2 | Ash et al., 2013, Sajjadi et al., 2012a* | 1/3 | Ash and Grossman, 2015; Fraser et al., 2015b*; Fraser et al., 2014a | 1/1 | Sajjadi et al., 2012a* | **3/6** |
|  |  | L/Pv = HC | 0/1 | Ash et al., 2013 | 0/1 | Ash and Grossman, 2015 | 0/0 | - | 0/2 |
|  |  | NF/av < Sv | 1/1 | Ash et al., 2013* | 1/1 | Fraser et al., 2014a* | 0/0 | - | **2/2** |
|  |  | NF/av = L/Pv | 0/1 | Ash et al., 2013 | 0/0 | - | 0/0 | - | 0/1 |
|  |  | Sv = L/Pv | 0/1 | Ash et al., 2013 |  |  | 0/0 | - | 0/1 |
|  | Max depth | NF/av < HC | 0/0 | - | 1/2 | Fraser et al., 2015b*; Fraser et al., 2014a | 0/0 | - | 1/2 |
|  |  | Sv < HC | 0/0 | - | 1/2 | Fraser et al., 2015b*; Fraser et al., 2014a | 0/0 | - | 1/2 |
|  |  | NF/av = Sv | 0/0 | - | 0/1 | Fraser et al., 2014a | 0/0 | - | 0/1 |
|  | **Mean depth** | NF/av < HC | 0/0 | - | 1/2 | Fraser et al., 2015b*; Fraser et al., 2014a | 0/0 | - | 1/2 |
|  |  | Sv < HC | 0/0 | - | 2/2 | Fraser et al., 2015b*; Fraser et al., 2014a* | 0/0 | - | **2/2** |
|  | **Total depth** | NF/av < HC | 0/0 | - | 1/2 | Fraser et al., 2015b*; Fraser et al., 2014a | 0/0 | - | 1/2 |
|  |  | Sv < HC | 0/0 | - | 2/2 | Fraser et al., 2015b*; Fraser et al., 2014a* | 0/0 | - | **2/2** |
|  | Tree height | NF/av < HC | 0/0 | - | 1/2 | Fraser et al., 2015b*; Fraser et al., 2014a | 0/0 | - | 1/2 |
|  |  | Sv < HC | 0/0 | - | 1/2 | Fraser et al., 2015b*; Fraser et al., 2014a | 0/0 | - | 1/2 |
|  | **Syntactic errors** | NF/av < HC | 1/1 | Wilson et al., 2010* | 0/0 | - | 1/1 | Knibb et al., 2009* | **2/2** |
|  |  | Sv = HC | 0/1 | Wilson et al., 2010 | 0/0 | - | 0/0 | - | 0/1 |
|  |  | L/Pv > HC | 1/1 | Wilson et al., 2010* | 0/0 | - | 0/0 | - | 1/1 |
|  |  | NF/av = Sv | 0/1 | Wilson et al., 2010 | 0/0 | - | 0/0 | - | 0/1 |
|  |  | NF/av = L/Pv | 0/1 | Wilson et al., 2010 | 0/0 | - | 0/0 | - | 0/1 |
|  |  | Sv < L/Pv | 1/1 | Wilson et al., 2010* | 0/0 | - | 0/0 | - | 1/1 |
| **DISCOURSE AND PRAGMATIC** | **Total words** | NF/av < HC | 3/3 | Ash et al., 2013*; Graham et al., 2004*; Wilson et al., 2010* | 2/2 | Ash and Grossman, 2015*; Fraser et al., 2014a* | 0/0 | - | **5/5** |
|  |  | Sv < HC | 1/3 | Ash et al., 2013*; Garrard and Forsyth, 2010, Wilson et al., 2010 | 0/2 | Ash and Grossman, 2015; Fraser et al., 2014a | 0/0 | - | 1/5 |
|  |  | L/Pv < HC | 1/2 | Ash et al., 2013*; Wilson et al., 2010 | 0/1 | Ash and Grossman, 2015 | 0/0 | - | 1/3 |
|  |  | NF/av < Sv | 1/1 | Ash et al., 2013* | 0/0 | - | 0/0 | - | 1/1 |
|  |  | NF/av < L/Pv | 1/1 | Ash et al., 2013* | 0/0 | - | 0/0 | - | 1/1 |
|  |  | SD = L/Pv | 0/1 | Ash et al., 2013 | 0/0 | - | 0/0 | - | 0/1 |
|  | Discourse markers (Fluency disruption) | NF/av > HC | 0/1 | Sajjadi et al., 2012b | 1/1 | Fraser et al., 2014b* | 0/1 | Sajjadi et al., 2012b | 1/3 |
|  |  | Sv > HC | 0/1 | Sajjadi et al., 2012a | 0/0 | - | 1/1 | Sajjadi et al., 2012a* | 1/2 |
|  | Cohesion | NF/av < HC | 0/0 | - | 1/1 | Fraser et al., 2014b* | 0/0 | - | 1/1 |
|  | **Local coherence** | NF/av < HC | 0/0 | - | 2/2 | Ash and Grossman, 2015*; Ash et al., 2006* | 0/0 | - | **2/2** |
|  |  | Sv < HC | 0/0 | - | 2/2 | Ash and Grossman, 2015*; Ash et al., 2006* | 0/0 | - | **2/2** |
|  |  | L/Pv < HC | 0/0 | - | 1/1 | Ash and Grossman, 2015*; | 0/0 | - | 1/1 |
|  | Global coherence | NF/av < HC | 0/0 | - | 1/2 | Ash and Grossman, 2015*; Ash et al., 2006 | 0/0 | - | 1/2 |
|  |  | Sv < HC | 0/0 | - | 1/2 | Ash and Grossman, 2015*; Ash et al., 2006 | 0/0 | - | 1/2 |
|  |  | L/Pv < HC | 0/0 | - | 1/1 | Ash and Grossman, 2015* | 0/0 | - | 1/1 |
|  | Implausible or irrelevant details | NF/av =HC | 0/1 | Graham et al., 2004 | 0/0 | - | 0/0 | - | 0/1 |
|  | Incomplete content elements | NF/av > HC | 0/0 | - | 1/1 | Ash et al., 2006* | 0/0 | - | 1/1 |
|  |  | Sv > HC | 0/0 | - | 1/1 | Ash et al., 2006* | 0/0 | - | 1/1 |
|  | Errors in content elements | NF/av = HC | 0/0 | - | 0/1 | Ash et al., 2006 | 0/0 | - | 0/1 |
|  |  | Sv > HC | 0/0 | - | 1/1 | Ash et al., 2006* | 0/0 | - | 1/1 |
|  |  | NF/av < Sv | 0/0 | - | 1/1 | Ash et al., 2006* | 0/0 | - | 1/1 |
|  | **Information content** | NF/av < HC | 1/1 | Sajjadi et al., 2012b* | 0/0 | - | 1/1 | Sajjadi et al., 2012b* | **2/2** |
|  |  | Sv < HC | 1/1 | Sajjadi et al., 2012a* | 0/0 | - | 1/1 | Sajjadi et al., 2012a* | **2/2** |
|  | **Topic maintenance**  (story narration: Frog story) | NF/av < HC | 0/0 | - | 2/2 | Ash and Grossman, 2015*; Ash et al., 2006* | 0/0 | - | **2/2** |
|  |  | Sv < HC | 0/0 | - | 1/2 | Ash and Grossman, 2015*; Ash et al., 2006 | 0/0 | - | 1/2 |
|  |  | L/Pv < HC | 0/0 | - | 1/1 | Ash and Grossman, 2015*; | 0/0 | - | 1/1 |
|  | **Information unit** (picture description) or **accurate report**  (story narration) | NF/av < HC | 2/2 | Graham et al., 2004*; Sajjadi et al., 2012b* | 2/2 | Ash and Grossman, 2015*; Ash et al., 2006* | 0/0 | - | **4/4** |
|  |  | Sv < HC | 1/1 | Sajjadi et al., 2012a* | 2/2 | Ash and Grossman, 2015*; Ash et al., 2006* | 0/0 | - | **3/3** |
|  |  | L/Pv < HC | 0/0 | - | 1/1 | Ash and Grossman, 2015* | 0/0 | - | 1/1 |
|  | **Spontaneity** | NF/av < HC | 1/1 | Sajjadi et al., 2012b* | 0/0 | - | 1/1 | Sajjadi et al., 2012b* | **2/2** |
|  |  | Sv < HC | 1/1 | Sajjadi et al., 2012a* | 0/0 | - | 0/1 | Sajjadi et al., 2012a | 1/2 |

Supplementary Table 3. Results of studies assessing linguistic variables in connected speech in Alzheimer’s disease and amnestic Mild Cognitive Impairment. The results are expressed as the ratio between the studies reporting significant between-group differences and the number of studies inquiring each specific feature. Dark gray rows represent variables significant in at least more than half of the studies considered for each variable (with at least three studies investigating the same feature) or in two out of two studies; the light grey rows represent the features that were tested and found significant in only one study. White rows represent variables not significant. An asterisk (*) is used to highlight the specific studies founding significant between-group differences.

AD = Alzheimer’s Disease;

HC = Healthy Controls;

aMCI = amnestic Mild Cognitive Impairment.

Studies including only patients in the early stages of Alzheimer’s disease are underlined.

| **LINGUISTIC LEVEL** | **FEATURE** | **RESULTS** | PICTURE DESCRIPTION  Ratio References | | STORY NARRATION  Ratio References | | INTERVIEW  Ratio References | | TOT  RATIO |
| --- | --- | --- | --- | --- | --- | --- | --- | --- | --- |
| **PHONETIC** | Skewness(MFCC) | AD ≠ HC | 1/2 | Fraser et al., 2015a*; Yancheva et al., 2015 | 0/0 | - | 0/0 | - | 1/2 |
|  | Kurtosis(MFCC) | AD ≠ HC | 1/2 | Fraser et al., 2015a*; Yancheva et al., 2015 | 0/0 | - | 0/0 | - | 1/2 |
|  | Total pause time | AD > HC | 0/1 | Yancheva et al., 2015 | 1/1 | Ash and Grossman, 2015* | 0/0 | - | 1/2 |
|  | Mean pauses duration | AD > HC | 0/1 | Yancheva et al., 2015 | 0/0 | - | 1/1 | Singh et al., 2001* | 1/2 |
|  | **Hesitation ratio** | AD > HC | 1/2 | Sajjadi et al., 2012a*; Cuetos et al., 2007 | 0/0 | - | 2/2 | Hoffmann et al., 2010*; Sajjadi et al., 2012a* | **3/4** |
|  | Filled pauses | AD = HC | 0/1 | Ahmed et al., 2013b | 0/0 | - | 0/0 | - | 0/1 |
|  | Speech tempo | AD < HC | 0/0 | - | 0/0 | - | 1/1 | Hoffmann et al., 2010* | 1/1 |
|  | Syllables per minutes | AD < HC | 0/0 | - | 0/0 | - | 1/1 | Guinn et al., 2012* | 1/1 |
|  | **Speech rate** | AD < HC | 2/3 | Carlomagno et al., 2005*; Sajjadi et al., 2012a*; Ahmed et al., 2013a | 2/2 | Ash et al., 2007*; Ash and Grossman, 2015* | 2/2 | Sajjadi et al., 2012a*; Singh et al., 2001* | **6/7** |
|  | Total phonation time | AD > HC | 0/1 | Fraser et al., 2015a | 0/0 | - | 1/1 | Singh et al., 2001* | 1/2 |
|  | Standardized phonation time | AD < HC | 0/0 | - | 0/0 | - | 1/1 | Singh et al., 2001* | 1/1 |
|  | Total locution time | AD > HC | 1/2 | Fraser et al., 2015a*; Ahmed et al., 2013a | 1/2 | Ash and Grossman, 2015*; Drummond et al 2015 | 0/1 | Singh et al., 2001 | 2/5 |
|  |  | aMCI = HC | 0/0 | - | 0/1 | Drummond et al., 2015 | 0/0 | - | 0/1 |
|  | Prosodic disruption | AD > HC | 1/2 | Forbes-McKay et al., 2013*; Forbes-McKay and Venneri, 2005 | 0/0 | - | 0/0 | - | 1/2 |
|  | F0 SD (prosody) | AD = HC | 0/1 | Yancheva et al., 2015 | 0/0 | - | 0/0 | - | 0/1 |
|  | Phonetic errors | AD = HC | 0/3 | Ahmed et al., 2013b; Forbes-Mckay et al., 2013; Forbes-McKay and Venneri, 2005 | 0/0 | - | 0/0 | - | 0/3 |
|  |  | AD = aMCI | 0/1 | Ahmed et al., 2013b | 0/0 | - | 0/0 | - | 0/1 |
| **PHONOLOGICAL** | Phonemic errors | AD > HC | 2/8 | Carlomagno et al., 2005*; Sajjadi et al., 2012a*; Ahmed et al., 2013b; Croisile et al., 1996; Cuetos et al., 2007; Forbes-Mckay et al., 2013; Forbes-McKay and Venneri, 2005; Nicholas et al., 1985 | 0/2 | Ash and Grossman, 2015; De Lira et al., 2010 | 1/2 | Sajjadi et al., 2012a*; Visch Brink et al., 2009 | 3/12 |
|  |  | AD = aMCI | 0/1 | Ahmed et al., 2013b | 0/0 | - |  |  | 0/1 |
|  | False starts | AD < HC | 1/3 | Sajjadi et al., 2012a*; Ahmed et al., 2013b; Cuetos et al., 2007 | 0/0 | - | 2/4 | Sajjadi et al., 2012a*; Guinn et al., 2012*; Ripich and Terrel, 1988; Visch Brink et al., 2009 | 3/7 |
|  |  | AD = aMCI | 0/1 | Ahmed et al., 2013b | 0/0 | - | 0/0 | - | 0/1 |
| **LEXICO-SEMANTIC** | Noun rate | AD < HC | 2/4 | Fraser et al., 2015a*; March et al., 2006*; Ahmed et al., 2013b; Croisile et al., 1996; | 1/2 | Ash and Grossman, 2015*; March et al., 2006* | 1/3 | Jarrold et al., 2014*; Bucks et al., 2000; Guinn et al., 2012 | 4/9 |
|  |  | AD = aMCI | 0/1 | Ahmed et al., 2013b | 0/0 | - | 0/0 | - | 0/1 |
|  | Verb rate | AD < HC | 2/4 | Fraser et al., 2015a*; Yancheva et al., 2015*; Ahmed et al., 2013b; Croisile et al., 1996; | 0/0 | - | 0/0 | - | 4/7 |
|  |  | AD > HC | 0/0 | - | 0/0 | - | 2/3 | Bucks et al., 2000*; Jarrold et al., 2014*; Guinn et al., 2012 |  |
|  |  | AD = aMCI | 0/1 | Ahmed et al., 2013b | 0/0 | - | 0/0 | - | 0/1 |
|  | Demonstrative rate | AD = HC | 0/1 | Fraser et al., 2015a | 0/0 | - | 0/0 | - | 0/1 |
|  | Adjective rate | AD > HC | 0/2 | Croisile et al., 1996; Fraser et al., 2015a; | 0/0 | - | 2/3 | Bucks et al., 2000*; Jarrold et al., 2014*; Guinn et al., 2012 | 2/5 |
|  | Pronoun rate | AD > HC | 1/2 | Ahmed et al., 2013b*; Fraser et al., 2015a; | 0/0 | - | 1/3 | Jarrold et al., 2014*; Bucks et al., 2000; Guinn et al., 2012 | 2/5 |
|  |  | AD > aMCI | 1/1 | Ahmed et al., 2013b* | 0/0 | - | 0/0 | - | 1/1 |
|  | Adverb rate | AD = HC | 0/2 | Croisile et al., 1996; Fraser et al., 2015a | 0/0 | - | 0/0 | - | 0/2 |
|  | Conjunction rate | AD > HC | 1/2 | Nicholas et al., 1985*; Fraser et al., 2015a | 0/0 | - | 0/1 | Ripich et al., 2000 | 1/3 |
|  | Noun ratio | AD = HC | 0/1 | Kavè and Levy, 2003 | 0/0 | - | 0/0 | - | 0/1 |
|  | **Pronoun-noun ratio** | AD > HC | 2/2 | Fraser et al., 2015a*; Kavè and Levy, 2003* | 0/0 | - | 0/0 | - | **2/2** |
|  | **Closed-class words** | AD > HC | 2/3 | Croisile et al., 1996*; Sajjadi et al., 2012a*; Orimaye et al., 2014 | 1/1 | Drummond et al., 2015* | 1/1 | Sajjadi et al., 2012a | **3/5** |
|  |  | aMCI = HC | 0/0 | - | 0/1 | Drummond et al., 2015 | 0/0 | - | 0/1 |
|  | Open class words | AD = HC | 0/0 | - | 0/1 | Drummond et al., 2015 | 0/0 | - | 0/1 |
|  |  | aMCI = HC | 0/0 | - | 0/1 | Drummond et al., 2015 | 0/0 | - | 0/1 |
|  | Content Density | AD = HC | 0/1 | Cuetos et al., 2007 | 0/0 | - | 0/0 | - | 0/1 |
|  | **Idea density** | AD < HC | 1/2 | Ahmed et al., 2013b*; Ahmed et al., 2013a | 0/0 | - | 1/1 | Jarrold et al., 2010* | **2/3** |
|  |  | AD < aMCI | 1/1 | Ahmed et al., 2013b* | 0/0 | - | 0/0 | - | 1/1 |
|  | Deictic terms | AD > HC | 1/1 | Nicholas et al., 1985* | 0/0 | - | 0/0 | - | 1/1 |
|  | Spatial deixis | AD > HC | 1/1 | March et al., 2006* | 0/1 | March et al., 2006 | 0/0 | - | 1/2 |
|  | Person deixis | AD < HC | 0/1 | March et al., 2006 | 1/1 | March et al., 2006* | 0/0 | - | 1/2 |
|  | Honoré’s statistic | AD < HC | 2/2 | Fraser et al., 2015a*; Yancheva et al., 2015*; | 0/0 | - | 0/2 | Bucks et al., 2000; Guinn et al., 2012 | 2/4 |
|  | Brunét’s index | AD > HC | 0/1 | Fraser et al., 2015a; | 0/0 | - | 1/2 | Bucks et al., 2000*; Guinn et al., 2012 | 1/3 |
|  | Type-token ratio | AD < HC | 0/3 | Fraser et al., 2015a; Orimaye et al., 2014; Yancheva et al., 2015 | 0/0 | - | 2/3 | Bucks et al., 2000*; Visch Brink et al., 2009*; Guinn et al., 2012 | 2/6 |
|  | **Frequency** | AD > HC | 2/2 | Fraser et al., 2015a*; Yancheva et al., 2015* | 0/0 | - | 0/0 | - | **2/2** |
|  | Familiarity | AD = HC | 0/2 | Fraser et al., 2015a; Yancheva et al., 2015 | 0/0 | - | 0/0 | - | 0/2 |
|  | Age of acquisition | AD = HC | 0/2 | Fraser et al., 2015a; Yancheva et al., 2015 | 0/0 | - | 0/0 | - | 0/2 |
|  | Word length | AD < HC | 1/2 | Fraser et al., 2015a*; Orimaye et al., 2014 | 0/0 | - | 0/0 | - | 1/2 |
|  | Open class word errors | AD > HC | 0/1 | Sajjadi et al., 2012a | 0/0 | - | 1/4 | Altmann et al., 2001*; Ripich et al., 2000; Ripich and Terrel,, 1988; Sajjadi et al., 2012a | 1/5 |
|  | Closed class word errors |  | 0/1 | Sajjadi et al., 2012a | 0/0 | - | 1/4 | Altmann et al., 2001*; Ripich et al., 2000; Ripich and Terrel,, 1988; Sajjadi et al., 2012a | 1/5 |
|  | **Semantic errors** | AD > HC | 5/6 | Forbes-McKay et al., 2013*; Forbes-McKay and Venneri, 2005*; Kavè and Levy, 2003*; Nicholas et al., 1985*; Sajjadi et al., 2012a*; Croisile et al., 1996, | 0/0 | - | 1/3 | Kempler et al., 1987*; Visch Brink et al., 2009, Sajjadi et al., 2012a | **6/9** |
|  | **Word-finding difficulties (lexical errors)** | AD > HC | 3/3 | Forbes-McKay et al., 2013*; Forbes-McKay and Venneri, 2005*; Croisile et al., 1996* | 2/2 | Ash et al., 2007*; De Lira et al., 2010* | 0/1 | Lai, 2014 | **5/6** |
|  | **Indefinite terms (lexical errors)** | AD > HC | 2/2 | Feyereisen et al., 2007*; Nicholas et al., 1985*; | 0/0 | - | 2/3 | Lai, 2014*; Visch Brink et al., 2009*; Dijkstra et al., 2004 | **4/5** |
|  | **Revision (lexical errors)** | AD > HC | 3/3 | Forbes-McKay et al., 2013*; Forbes-McKay and Venneri, 2005*; Orimaye et al., 2014*; | 0/0 | - | 0/0 | - | **3/3** |
|  | **Repetitions (lexical errors)** | AD > HC | 3/4 | Sajjadi et al., 2012a*; Nicholas et al., 1985*; Orimaye et al., 2014*; Croisile et al., 1996 | 2/2 | Drummond et al 2015*; De Lira et al., 2010* | 3/5 | Guinn et al., 2012*; Sajjadi et al., 2012a*; Visch Brink et al., 2009*; Lai, 2014, Dijkstra et al., 2004 | **8/11** |
|  |  | aMCI > HC | 0/0 | - | 1/1 | Drummond et al 2015* | 0/0 | - | 1/1 |
|  | Perseveration | AD = aMCI | 0/1 | Ahmed et al., 2013b | 0/0 | - | 0/0 | - | 0/1 |
|  | Repaired sequences | AD > HC | 0/2 | Ahmed et al., 2013b; Croisile et al., 1996 | 1/2 | De Lira et al., 2010*; Ash and Grossman, 2015 | 1/2 | Lai, 2014*; Visch Brink et al., 2009 | 2/6 |
|  |  | AD = aMCI | 0/1 | Ahmed et al., 2013b | 0/0 | - | 0/0 | - | 0/1 |
|  | **Response to word finding delays** | AD < HC | 2/2 | Forbes-McKay et al., 2013*; Forbes-McKay and Venneri, 2005*; | 0/0 | - | 0/0 | - | **2/2** |
|  | Not in dictionary (lexical errors) | AD > HC | 2/3 | Fraser et al., 2015a*; Yancheva et al., 2015*; Nicholas et al., 1985 | 0/0 | - | 0/1 | Visch Brink et al., 2009 | 2/4 |
|  | Circumlocutory comments | AD > HC | 1/4 | Kavè and Levy, 2003*; Cuetos et al., 2007; Nicholas et al., 1985; Sajjadi et al., 2012a | 0/0 | - | 0/1 | Sajjadi et al., 2012a | 1/5 |
| **MORPHOSYNTACTIC** | Inflected verbs | AD < HC | 1/4 | Fraser et al., 2015a*; Ahmed et al., 2013b; Cuetos et al., 2007; Kavè and Levy, 2003 | 0/1 | Ash and Grossman, 2015, | 0/0 | - | 1/5 |
|  |  | AD = aMCI | 0/1 | Ahmed et al., 2013b | 0/0 | - |  |  | 1/1 |
|  | Subject-verb agreement | AD < HC | 0/0 | - | 0/0 | - | 1/1 | Kaprinis et al., 2007* | 1/1 |
|  | Correct verb tense | AD < HC | 0/0 | - | 0/0 | - | 1/2 | Dijkstra et al., 2004*; Kaprinis et al., 2007 | 1/2 |
|  | Auxiliary verbs | AD < HC | 1/2 | Fraser et al., 2015a*; Cuetos et al., 2007 | 0/0 | - | 0/0 | - | 1/2 |
|  | Gerunds | AD < HC | 1/1 | Fraser et al., 2015a* | 0/0 | - | 0/0 | - | 1/1 |
|  | Participles | AD < HC | 1/1 | Fraser et al., 2015a* | 0/0 | - | 0/0 | - | 1/1 |
|  | Object clitics | AD < HC |  |  | 0/0 | - | 1/1 | Kaprinis et al., 2007* | 1/1 |
|  | Grammatical form | AD < HC | 1/3 | Forbes-Mckay et al., 2013*; Forbes-McKay and Venneri, 2005; Croisile et al., 1996 | 0/0 | - | 1/2 | Hoffmann et al., 2010; Lai, 2014* | 2/5 |
|  | **Inflectional errors** | AD > HC | 1/2 | Sajjadi et al., 2012a*; Cuetos et al., 2007 | 0/0 | - | 1/2 | Sajjadi et al., 2012a, Altmann et al., 2001* | **2/4** |
| **SYNTACTIC** | Clauses | AD = HC | 0/3 | Croisile et al., 1996; Fraser et al., 2015a; Sajjadi et al., 2012a | 0/0 | - | 0/1 | Sajjadi et al., 2012a | 0/4 |
|  | T-units | AD = HC | 0/1 | Fraser et al., 2015a | 0/0 | - | 0/0 | - | 0/1 |
|  | Sentences | AD = HC | 0/2 | Cuetos et al., 2007; Orimaye et al., 2014 | 0/0 | - | 0/0 | - | 0/2 |
|  | Utterances | AD > HC | 1/1 | Orimaye et al., 2014* | 0/1 | Ash and Grossman, 2015 | 0/1 | Ripich et al., 2000 | 1/3 |
|  | **Mean length of utterance** | AD < HC | 2/6 | Orimaye et al., 2014*; Yancheva et al., 2015*; Ahmed et al., 2013b, Cuetos et al., 2007, Forbes-Mckay et al., 2013, Fraser et al., 2015a | 2/2 | Ash and Grossman, 2015*; Ash et al., 2007* | 0/1 | Ripich et al., 2000 | **7/12** |
|  | **Mean length of clause** | AD > HC | 1/1 | Kavè and Levy, 2003* | 0/0 | - | 0/0 | - |  |
|  | **Mean length of T-unit** | AD < HC | 1/1 | Sajjadi et al., 2012a* | 0/0 | - | 1/1 | Sajjadi et al., 2012a* |  |
|  | Mean length of sentence | AD = aMCI | 0/1 | Ahmed et al., 2013b | 0/0 | - | 0/0 | - | 0/1 |
|  | Incomplete sentences  (Fluency disruption) | AD > HC | 1/5 | Sajjadi et al., 2012a*; Ahmed et al., 2013b; Croisile et al., 1996; Cuetos et al., 2007; Kavè and Levy, 2003 | 0/0 | - | 2/3 | Ripich and Terrel, 1988*; Sajjadi et al., 2012a*; Dijkstra et al., 2004 | 3/8 |
|  |  | AD = aMCI | 0/1 | Ahmed et al., 2013b | 0/0 | - | 0/0 | - | 0/1 |
|  | **Reduced sentences** | AD > HC | 2/2 | Orimaye et al., 2014*; Sajjadi et al., 2012a* | 1/1 | De Lira et al., 2010* | 1/1 | Sajjadi et al., 2012a* | **4/4** |
|  | Well-formed sentences | AD < HC | 0/0 |  | 1/1 | Ash and Grossman, 2015* | 0/1 | Visch Brink et al., 2009 | 1/2 |
|  | Nouns with determiner | AD = HC | 0/1 | Ahmed et al., 2013b | 0/0 | - | 0/0 | - | 0/1 |
|  |  | AD = aMCI | 0/1 | Ahmed et al., 2013b | 0/0 | - | 0/0 | - | 0/1 |
|  | Noun phrases | AD < HC | 1/1 | Yancheva et al., 2015* | 0/0 | - | 0/0 | - | 1/1 |
|  | Verb phrases | AD < HC | 2/2 | Orimaye et al., 2014*; Yancheva et al., 2015 | 0/0 | - | 0/0 | - | 2/2 |
|  |  | AD < HC | 1/1 | Yancheva et al., 2015* | 0/0 | - | 0/0 | - | 1/1 |
|  | Embeddings | AD = HC | 0/1 | Ahmed et al., 2013b | 0/0 | - | 0/1 | Kaprinis et al., 2007 | 0/2 |
|  |  | AD = aMCI | 0/1 | Ahmed et al., 2013b | 0/0 | - | 0/0 | - | 0/1 |
|  | Coordinate sentences | AD < HC | 0/2 | Orimaye et al., 2014; Yancheva et al., 2015 | 1/1 | De Lira et al., 2010* | 0/0 | - | 1/3 |
|  | Dependent clauses | AD < HC | 2/5 | Croisile et al., 1996*; Sajjadi et al., 2012a*; Kavè and Levy, 2003; Orimaye et al., 2014; Yancheva et al., 2015 | 0/2 | Ash and Grossman, 2015; De Lira et al., 2010 | 2/4 | Visch Brink et al., 2009*; Sajjadi et al., 2012a*; Kaprinis et al., 2007; Ripich et al., 2000 | 4/11 |
|  | Empty phrases | AD > HC | 1/1 | Nicholas et al., 1985* | 0/0 | - | 1/4 | Guinn et al., 2012*; Lai, 2014; Visch Brink et al., 2009; Dijkstra et al., 2004 | 2/5 |
|  | Max depth | AD = HC | 0/1 | Yancheva et al., 2015 | 0/0 | - | 0/0 | - | 0/1 |
|  | Mean depth | AD = HC | 0/2 | Fraser et al., 2015a; Yancheva et al., 2015 | 0/0 | - | 0/0 | - | 0/2 |
|  | Total depth | AD = HC | 0/2 | Fraser et al., 2015a; Yancheva et al., 2015 | 0/0 | - | 0/0 | - | 0/2 |
|  | Tree height | AD = HC | 0/2 | Fraser et al., 2015a; Yancheva et al., 2015 | 0/0 | - | 0/0 | - | 0/2 |
|  |  | AD < HC |  |  | 0/0 | - | 1/1 | Lai, 2014* | 1/1 |
|  | Syntactic errors | AD = HC | 0/1 | Ahmed et al., 2013b | 0/0 | - | 0/0 | - | 0/1 |
|  |  | AD = aMCI | 0/1 | Ahmed et al., 2013b | 0/0 | - | 0/0 | - | 0/1 |
| **DISCOURSE AND PRAGMATIC** | Total words | AD < HC | 1/10 | Croisile et al., 1996*; Ahmed et al., 2013a; Bschor et al., 2001; Carlomagno et al., 2003; Feyereisen et al., 2007; Fraser et al., 2015a; Nicholas et al., 1985; Orimaye et al., 2014; Shimada et al., 1998; Sajjadi et al., 2012a | 0/2 | Ash and Grossman, 2015; Drummond et al 2015 | 0/3 | Ripich et al., 2000; Singh et al., 2001; Sajjadi et al., 2012a | 2/16 |
|  |  | AD > HC |  |  |  |  | 1/1 | Ripich and Terrel, 1988* |  |
|  |  | AD = aMCI | 0/1 | Ahmed et al., 2013b; Bschor et al., 2001 | 0/0 | - | 0/0 | - | 0/1 |
|  | **Discourse markers (Fluency disruption)** | AD > HC | 0/1 | Sajjadi et al., 2012a | 0/0 | - | 3/3 | Visch Brink et al., 2009*; Sajjadi et al., 2012a*; Guinn et al., 2012* | **3/3** |
|  | **Cohesion** | AD < HC | 0/0 | - | 1/1 | Drummond et al 2015* | 1/1 | Lai, 2014* | **2/2** |
|  |  | aMCI = HC | 0/0 | - | 0/1 | Drummond et al 2015 | 0/0 | - | 0/1 |
|  | **Correct pronoun** | AD < HC | 1/1 | Nicholas et al., 1985* | 0/0 | - | 4/4 | Altmann et al., 2001*; Lai, 2014*; Ripich et al., 2000*; Dijkstra et al., 2004* | **5/5** |
|  | **Local coherence** | AD < HC | 0/0 | - | 2/2 | Ash and Grossman, 2015*; Ash et al., 2007* | 1/2 | Lai, 2014*; Dijkstra et al., 2004 | **3/4** |
|  | **Global coherence** | AD < HC | 0/0 | - | 1/1 | Ash and Grossman, 2015* | 2/2 | Lai, 2014*; Dijkstra et al., 2004* | **3/3** |
|  | Microproposition (coherence) story narration | AD > HC | 0/0 | - | 1/1 | Drummond et al 2015* | 0/0 | - | 1/1 |
|  |  | aMCI > HC | 0/0 | - | 1/1 | Drummond et al 2015* | 0/0 | - | 1/1 |
|  | **Implausible or irrelevant details** | AD > HC | 2/2 | Carlomagno et al., 2003*; Croisile et al., 1996* | 1/1 | Drummond et al 2015* | 0/0 | - | **3/3** |
|  |  | aMCI > HC | 0/0 | - | 1/1 | Drummond et al 2015* | 0/0 | - | 1/1 |
|  | Index of discourse effectiveness | AD < HC | 0/0 | - | 1/1 | Drummond et al 2015* | 0/0 | - | 1/1 |
|  |  | aMCI < HC | 0/0 | - | 1/1 | Drummond et al 2015* | 0/0 | - | 1/1 |
|  |  | AD < aMCI | 0/0 | - | 1/1 | Drummond et al 2015* | 0/0 | - | 1/1 |
|  | **Incomplete content elements** | AD > HC | 0/0 | - | 1/1 | Drummond et al 2015* | 1/1 | Dijkstra et al., 2004* | **2/2** |
|  |  | aMCI > HC | 0/0 | - | 1/1 | Drummond et al 2015* | 0/0 | - | 1/1 |
|  | Errors in content elements | AD > HC | 1/1 | Carlomagno et al., 2003* |  |  |  |  | 1/1 |
|  | **Information content** | AD < HC | 5/5 | Forbes-Mckay et al., 2013*; Forbes-McKay and Venneri, 2005*; Sajjadi et al., 2012a*; Carlomagno et al., 2003*; Cuetos et al., 2007* | 2/2 | Drummond et al 2015*; Ash et al., 2007* | 2/2 | Sajjadi et al., 2012a*; Dijkstra et al., 2004* | **9/9** |
|  |  | AD < aMCI | 0/0 | - | 1/1 | Drummond et al., 2015* | 0/0 | - | 1/1 |
|  | Ratio words/information | AD > HC | 1/2 | Croisile et al., 1996*; Shimada et al., 1998 | 0/0 | - | 0/0 | - | 1/2 |
|  | **Topic maintenance**  (story narration: Frog story) | AD < HC | 0/0 | - | 2/2 | Ash and Grossman, 2015*; Ash et al., 2007* | 1/1 | - Dijkstra et al., 2004* | **3/3** |
|  | **Information unit** (picture description) **or accurate report** (story narration) | AD < HC | 13/13 | Ahmed et al., 2013a*; Ahmed et al., 2013b*; Carlomagno et al., 2003*; Croisile et al., 1996*; Cuetos et al., 2007*; Feyereisen et al., 2007* Forbes-Mckay et al., 2013*. Forbes-McKay and Venneri, 2005*; Fraser et al., 2015a*; Kavè and Levy, 2003*; Nicholas et al., 1985*; Shimada et al., 1998*; Sajjadi et al., 2012a* | 1/1 | Ash and Grossman, 2015* | 1/1 | Lai, 2014* | **15/15** |
|  |  | AD < aMCI | 1/2 | Ahmed et al., 2013b*; Bschor et al., 2001 | 0/0 | - | 0/0 | - | 1/2 |
|  | **Subjects**  (picture description) | AD < HC | 3/4 | Ahmed et al., 2013a*; Ahmed et al., 2013b*; Bschor et al., 2001*; Kavè and Levy, 2003 | 0/0 | - | 0/0 | - | **3/4** |
|  |  | AD = aMCI | 0/2 | Ahmed et al., 2013b; Bschor et al., 2001 | 0/0 | - | 0/0 | - | 0/2 |
|  | Objects  (picture description) | AD < HC | 2/4 | Bschor et al., 2001*; Kavè and Levy, 2003*; Ahmed et al., 2013a, Ahmed et al., 2013b | 0/0 | - | 0/0 | - | 2/4 |
|  |  | AD < aMCI | 1/2 | Ahmed et al., 2013b*; Bschor et al., 2001 | 0/0 | - | 0/0 | - | 1/2 |
|  | Places  (picture description) | AD < HC | 1/3 | Bschor et al., 2001*; Ahmed et al., 2013a; Kavè and Levy, 2003 | 0/0 | - | 0/0 | - | 1/3 |
|  |  | AD = aMCI | 0/2 | Ahmed et al., 2013b; Bschor et al., 2001 | 0/0 | - | 0/0 | - | 0/2 |
|  | **Actions**  (picture description) | AD < HC | 3/4 | Ahmed et al., 2013a*; Bschor et al., 2001*; Kavè and Levy, 2003*; Ahmed et al., 2013b | 0/0 | - | 0/0 | - | **3/4** |
|  |  | AD = aMCI | 0/2 | Ahmed et al., 2013b; Bschor et al., 2001 | 0/0 | - | 0/0 | - | 0/2 |
|  | Subject+places+objects  (picture description) | AD < HC | 1/1 | Ahmed et al., 2013a* | 0/0 | - | 0/0 | - | 1/1 |
|  |  | AD = aMCI | 0/1 | Ahmed et al., 2013a | 0/0 | - | 0/0 | - | 0/1 |
|  | Info_window (picture description: Cookies Theft) | AD < HC | 1/1 |  | 0/0 | - | 0/0 | - | 1/1 |
|  | **Efficiency**  (picture description) | AD < HC | 2/2 | Ahmed et al., 2013a*; Ahmed et al., 2013b* | 0/0 | - | 0/0 | - | **2/2** |
|  |  | AD < aMCI | 1/1 | Ahmed et al., 2013a | 0/0 | - | 0/0 | - | 1/1 |
|  | Ration words/information unit | AD < HC | 0/1 | Carlomagno et al., 2003 | 0/0 | - | 1/1 | Dijkstra et al., 2004* | 1/2 |
|  | Modalizations | AD > HC | 1/2 | Croisile et al., 1996*; Nicholas et al., 1985 | 0/0 | - | 0/1 | Visch Brink et al., 2009 | 1/3 |
|  | Self-referential tags | AD > HC | 0/0 | - | 0/0 | - | 1/1 | Asp et al., 2006* | 1/1 |
|  |  | AD < HC | 0/0 | - | 0/0 | - | 1/1 | Guinn et al., 2012* | 1/1 |
|  | Paraphrasing | AD < HC | 0/0 | - | 0/0 | - | 1/1 | Guinn et al., 2012* | 1/1 |
|  | Spontaneity | AD = HC | 0/1 | Sajjadi et al., 2012a | 0/0 | - | 0/1 | Sajjadi et al., 2012a | 0/2 |

Supplementary Table 4. Results of studies assessing linguistic variables in connected speech in extrapyramidal movement disorders. The results are expressed as the ratio between the studies reporting significant between-group differences and the number of studies inquiring each specific feature. Dark gray rows represent variables significant in at least more than half of the studies considered for each variable (with at least three studies investigating the same feature) or in two out of two studies; the light grey rows represent the features that were tested and found significant in only one study. White rows represent variables not significant. An asterisk (*) is used to highlight the specific studies founding significant between-group differences

HC = Healthy Controls;

CBS = Corticobasal Syndrome

HD = Huntington’s Disease;

LBD = Dementia with Lewy Body;

PD = Parkinson’s Disease;

PDD = Parkinson’s Disease with Dementia.

| **LINGUISTIC LEVEL** | **FEATURE** | **RESULTS** | PICTURE DESCRIPTION  Ratio References | | STORY NARRATION  Ratio References | | INTERVIEW  Ratio References | | TOTAL  RATIO |
| --- | --- | --- | --- | --- | --- | --- | --- | --- | --- |
| **PHONETIC** | Number of pauses | PD > HC | 0/0 | - | 0/0 | - | 1/1 | Rusz et al., 2011 | 1/1 |
|  | Total pause time | PD > HC | 0/0 | - | 1/1 | Ash and Grossman, 2015* | 0/1 | Rusz et al., 2011 | 1/2 |
|  |  | PDD > HC | 0/0 | - | 1/1 | Ash and Grossman, 2015* | 0/0 | - | 1/1 |
|  |  | LBD > HC | 0/0 | - | 1/1 | Ash and Grossman, 2015* | 0/0 | - | 1/1 |
|  | Between –utterance pause duration | PD > HC | 0/0 | - | 1/1 | Ash et al., 2012b* | 0/0 | - | 1/1 |
|  |  | PDD > HC | 0/0 | - | 1/1 | Ash et al., 2012b* | 0/0 | - | 1/1 |
|  |  | LBD > HC | 0/0 | - | 1/1 | Ash et al., 2012b* | 0/0 | - | 1/1 |
|  | **Speech rate** | PD = HC | 0/0 | - | 0/3 | Ash and Grossman, 2015; Ash et al., 2012b; Ash et al., 2011 | 0/0 | - | 0/3 |
|  |  | PDD < HC | 0/0 | - | 3/3 | Ash and Grossman, 2015*; Ash et al., 2011*; Ash et al., 2012b*; | 0/0 | - | **3/3** |
|  |  | LBD < HC | 0/0 | - | 3/3 | Ash and Grossman, 2015*; Ash et al., 2011*; Ash et al., 2012b*; | 0/0 | - | **3/3** |
|  |  | PDD > LBD | 0/0 | - | 2/2 | Ash et al., 2011*; Ash et al., 2012b*; | 0/0 | - | **2/2** |
|  |  | PD > PDD | 0/0 | - | 1/1 | Ash et al., 2011* | 0/0 | - | 1/1 |
|  |  | PD > LBD | 0/0 | - | 1/1 | Ash et al., 2011* | 0/0 | - | 1/1 |
|  |  | CBS < HC | 0/0 | - | 1/1 | Gross et al., 2010* | 0/0 | - | 1/1 |
|  | Standardize phonation time | PDD > LBD | 0/0 | - | 1/1 | Ash et al., 2012b* | 0/0 | - | 1/1 |
|  | Total locution time | PD = HC | 0/0 | - | 0/1 | Ash and Grossman, 2015 | 0/0 | - | 0/1 |
|  |  | PDD = HC | 0/0 | - | 0/1 | Ash and Grossman, 2015 | 0/0 | - | 0/1 |
|  |  | LBD = HC | 0/0 | - | 0/1 | Ash and Grossman, 2015 | 0/0 | - | 0/1 |
|  | Prosody: F0 SD | PD < HC | 0/0 | - | 0/0 | - | 1/1 | Rusz et al., 2011* | 1/1 |
|  | Prosody: Intensity SD | PD < HC | 0/0 | - | 0/0 | - | 1/1 | Rusz et al., 2011* | 1/1 |
|  | **Phonetic errors** | PD = HC | 0/0 | - | 0/2 | Ash et al., 2012b; Ash et al., 2011 | 0/0 | - | 0/2 |
|  |  | PDD > HC | 0/0 | - | 2/2 | Ash et al., 2012b*; Ash et al., 2011* | 0/0 | - | **2/2** |
|  |  | LBD > HC | 0/0 | - | 2/2 | Ash et al., 2012b*; Ash et al., 2011* | 0/0 | - | **2/2** |
|  |  | PD < PDD | 0/0 | - | 1/1 | Ash et al., 2011* | 0/0 | - | 1/1 |
|  |  | PD < LBD | 0/0 | - | 1/1 | Ash et al., 2011* | 0/0 | - | 1/1 |
| **PHONOLOGICAL** | **Phonemic errors** | PD = HC | 0/0 | - | 0/2 | Ash and Grossman, 2015; Ash et al., 2011 | 0/0 | - | 0/2 |
|  |  | PDD > HC | 0/0 | - | 2/2 | Ash and Grossman, 2015*; Ash et al., 2011* | 0/0 | - | **2/2** |
|  |  | LBD > HC | 0/0 | - | 2/2 | Ash and Grossman, 2015*; Ash et al., 2011* | 0/0 | - | **2/2** |
|  |  | PD < PDD | 0/0 | - | 1/1 | Ash et al., 2011* | 0/0 | - | 1/1 |
|  |  | PD < LBD | 0/0 | - | 1/1 | Ash et al., 2011* | 0/0 | - | 1/1 |
| **LEXICO-SEMANTIC** | Noun rate | PD = HC | 0/0 | - | 0/1 | Ash and Grossman, 2015 | 0/0 | - | 0/1 |
|  |  | PDD = HC | 0/0 | - | 0/1 | Ash and Grossman, 2015 | 0/0 | - | 0/1 |
|  |  | LBD = HC | 0/0 | - | 0/1 | Ash and Grossman, 2015 | 0/0 | - | 0/1 |
|  |  | HD = HC | 0/1 | Jensen et al., 2006 | 0/0 | - | 0/0 | - | 0/1 |
|  | Verb rate | HD = HC | 0/1 | Jensen et al., 2006 | 0/0 | - | 0/0 | - | 0/1 |
|  | Adjective rate | HD = HC | 0/1 | Jensen et al., 2006 | 0/0 | - | 0/0 | - | 0/1 |
|  | Adverb rate | HD = HC | 0/1 | Jensen et al., 2006 | 0/0 | - | 0/0 | - | 0/1 |
|  | Closed-class words | PD = HC | 0/0 | - | 0/0 | - | 0/1 | Murray and Lenz, 2001 | 0/1 |
|  |  | HD = HC | 0/1 | Jensen et al., 2006 | 0/0 | - | 0/1 | Murray and Lenz, 2001 | 0/2 |
|  | Open class words | PD = HC | 0/0 | - | 0/2 | Ash et al., 2012b; Ash et al., 2011 | 0/0 | - | 0/2 |
|  |  | PDD = HC | 0/0 | - | 0/2 | Ash et al., 2012b; Ash et al., 2011 | 0/0 | - | 0/2 |
|  |  | LBD = HC | 0/0 | - | 0/2 | Ash et al., 2012b; Ash et al., 2011 | 0/0 | - | 0/2 |
|  | Word-finding difficulties (lexical errors) | HD = HC | 0/1 | Jensen et al., 2006 |  |  | 0/0 | - | 0/1 |
|  | Revision  (lexical errors) | PD = HC | 0/0 | - | 0/2 | Ash and Grossman, 2015; Ash et al., 2012b | 0/0 | - | 0/2 |
|  |  | PDD = HC | 0/0 | - | 0/2 | Ash and Grossman, 2015; Ash et al., 2012b | 0/0 | - | 0/2 |
|  |  | LBD = HC | 0/0 | - | 0/2 | Ash and Grossman, 2015; Ash et al., 2012b | 0/0 | - | 0/2 |
|  |  | HD = HC | 0/1 | Jensen et al., 2006 | 0/0 | - | 0/0 | - | 0/1 |
| **MORPHO-SYNTACTIC** | Inflected verbs | PD = HC | 0/0 | - | 0/1 | Ash and Grossman, 2015 | 0/1 | Murray and Lenz, 2001 | 0/2 |
|  |  | PDD = HC | 0/0 | - | 0/1 | Ash and Grossman, 2015 |  |  |  |
|  |  | LBD = HC | 0/0 | - | 0/1 | Ash and Grossman, 2015 |  |  |  |
|  |  | HD = HC | 0/0 | - | 0/0 | - | 0/1 | Murray and Lenz, 2001 | 0/1 |
|  | Grammatical form | PD = HC | 0/0 | - | 0/1 | Ash et al., 2012b | 0/0 | - | 0/1 |
|  |  | PDD = HC | 0/0 | - | 0/1 | Ash et al., 2012b | 0/0 | - | 0/1 |
|  |  | LBD < HC | 0/0 | - | 1/1 | Ash et al., 2012b* | 0/0 | - | 1/1 |
| **SYNTACTIC** | Clauses | HD = HC | 0/1 | Jensen et al., 2006 | 0/0 | - | 0/0 | - | 0/1 |
|  | Utterances | PD = HC | 0/0 | - | 0/1 | Ash and Grossman, 2015 | 0/0 | - | 0/1 |
|  |  | PDD < HC | 0/0 | - | 1/1 | Ash and Grossman, 2015* | 0/0 | - | 1/1 |
|  |  | LBD < HC | 0/0 | - | 1/1 | Ash and Grossman, 2015* | 0/0 | - | 1/1 |
|  |  | HD < HC | 0/0 | - |  |  | 1/1 | Murray and Lenz, 2001* | 1/1 |
|  | Mean length of utterance | PD = HC | 0/0 | - | 0/2 | Ash and Grossman, 2015; Ash et al., 2011 | 0/1 | Murray and Lenz, 2001 | 0/3 |
|  |  | PDD = HC | 0/0 | - | 0/2 | Ash and Grossman, 2015; Ash et al., 2011 | 0/0 | - | 0/2 |
|  |  | LBD = HC | 0/0 | - | 0/2 | Ash and Grossman, 2015; Ash et al., 2011 | 0/0 | - | 0/2 |
|  | Incomplete sentences (Fluency disruption) | HD = HC | 0/1 | Jensen et al., 2006 | 0/0 | - | 0/0 | - | 0/1 |
|  | **Well-formed sentences** | PD = HC | 0/0 | - | 0/3 | Ash and Grossman, 2015; Ash et al., 2012b; Ash et al., 2011 | 0/1 | Murray and Lenz, 2001 | 0/3 |
|  |  | PDD < HC | 0/0 | - | 2/3 | Ash and Grossman, 2015*; Ash et al., 2011*; Ash et al., 2012b | 0/0 | - | **2/3** |
|  |  | LBD < HC | 0/0 | - | 3/3 | Ash and Grossman, 2015*; Ash et al., 2011*; Ash et al., 2012b* | 0/0 | - | **3/3** |
|  |  | PD > PDD | 0/0 | - | 1/1 | Ash et al., 2011* | 0/0 | - | 1/1 |
|  |  | PD > LBD | 0/0 | - | 2/2 | Ash et al., 2011*; Ash et al., 2012b* | 0/0 | - | **2/2** |
|  |  | PDD > LBD | 0/0 | - | 1/1 | Ash et al., 2012b* |  |  | 1/1 |
|  |  | HD < HC | 0/0 | - | 0/0 | - | 1/1 | Murray and Lenz, 2001* | 1/1 |
|  | Embeddings | PD = HC | 0/0 | - | 0/0 | - | 0/1 | Murray and Lenz, 2001 | 0/1 |
|  |  | HD = HC | 0/0 | - | 0/0 | - | 0/1 | Murray and Lenz, 2001 | 0/1 |
|  | **Dependent clauses** | PD = HC | 0/0 | - | 0/3 | Ash and Grossman, 2015; Ash et al., 2012b; Ash et al., 2011 | 0/1 | Murray and Lenz, 2001 | 0/3 |
|  |  | PDD < HC | 0/0 | - | 1/3 | Ash et al., 2011*; Ash and Grossman, 2015; Ash et al., 2012b | 0/0 | - | 1/3 |
|  |  | LBD < HC | 0/0 | - | 2/3 | Ash et al., 2011*; Ash et al., 2012b*; Ash and Grossman, 2015 | 0/0 | - | **2/3** |
|  |  | PD > LBD | 0/0 | - | 1/1 | Ash et al., 2012b* | 0/0 | - | 1/1 |
|  |  | PDD > LBD | 0/0 | - | 1/1 | Ash et al., 2012b* | 0/0 | - | 1/1 |
|  |  | HD = HC | 0/1 | Jensen et al., 2006 | 0/0 | - | 0/1 | Murray and Lenz, 2001 | 0/2 |
|  | Syntactic errors | HD > HC | 1/1 | Jensen et al., 2006* | 0/0 | - | 0/0 | - | 1/1 |
| **DISCOURSE AND PRAGMATIC** | **Total words** | PD = HC | 0/0 | - | 0/3 | Ash and Grossman, 2015; Ash et al., 2012b; Ash et al., 2011 | 0/0 | - | 0/3 |
|  |  | PDD < HC | 0/0 | - | 3/3 | Ash and Grossman, 2015*; Ash et al., 2011*; Ash et al., 2012b* | 0/0 | - | **3/3** |
|  |  | LBD < HC | 0/0 | - | 3/3 | Ash and Grossman, 2015*; Ash et al., 2011*; Ash et al., 2012b* | 0/0 | - | **3/3** |
|  |  | PD > PDD | 0/0 | - | 1/1 | Ash et al., 2011* | 0/0 | - | 1/1 |
|  |  | PD > LBD | 0/0 | - | 1/1 | Ash et al., 2011* | 0/0 | - | 1/1 |
|  |  | HD < HC | 0/1 | Jensen et al., 2006 | 0/0 | - | 0/0 | - | 1/1 |
|  | **Local coherence** | PD < HC |  |  | 1/3 | Ash et al., 2012a*; Ash et al., 2011; Ash and Grossman, 2015 | 0/0 | - | 1/3 |
|  |  | PDD < HC | 0/0 | - | 3/3 | Ash and Grossman, 2015*; Ash et al., 2012a*; Ash et al., 2011* | 0/0 | - | **3/3** |
|  |  | LBD < HC | 0/0 | - | 3/3 | Ash and Grossman, 2015*; Ash et al., 2012a*; Ash et al., 2011* | 0/0 | - | **3/3** |
|  |  | PDD > LBD | 0/0 | - | 1/1 | Ash et al., 2011* | 0/0 | - | 1/1 |
|  |  | CBS < HC | 0/0 | - | 1/1 | Gross et al., 2010* | 0/0 | - | 1/1 |
|  | **Global coherence** | PD = HC | 0/0 | - | 0/2 | Ash and Grossman, 2015; Ash et al., 2011 | 0/0 | - | 0/2 |
|  |  | PDD < HC | 0/0 | - | 2/2 | Ash and Grossman, 2015*; Ash et al., 2011* | 0/0 | - | **2/2** |
|  |  | LBD < HC | 0/0 | - | 2/2 | Ash and Grossman, 2015*; Ash et al., 2011* | 0/0 | - | **2/2** |
|  |  | CBS < HC | 0/0 | - | 1/1 | Gross et al., 2010* | 0/0 | - | 1/1 |
|  | Implausible or irrelevant details | HD = HC | 0/1 | Jensen et al., 2006 | 0/0 | - | 0/0 | - | 0/1 |
|  | Information content | CBS < HC | 0/0 | - | 1/1 | Gross et al., 2010* | 0/0 | - | 1/1 |
|  | **Topic maintenance**  (story narration: Frog story) | CBS < HC | 0/0 | - | 1/1 | Gross et al., 2010* | 0/0 | - | 1/1 |
|  |  | PD < HC | 0/0 | - | 1/3 | Ash et al., 2012a*; Ash and Grossman, 2015; Ash et al., 2011 | 0/0 | - | 1/3 |
|  |  | PDD < HC | 0/0 | - | 3/3 | Ash and Grossman, 2015*; Ash et al., 2012a*; Ash et al., 2011* | 0/0 | - | **3/3** |
|  |  | LBD < HC | 0/0 | - | 3/3 | Ash and Grossman, 2015*; Ash et al., 2012a*; Ash et al., 2011* | 0/0 | - | **3/3** |
|  |  | PDD > LBD | 0/0 | - | 1/1 | Ash et al., 2011* | 0/0 | - | 1/1 |
|  | Information unit (picture description) or accurate report (story narration) | PD = HC | 0/0 | - | 0/1 | Ash and Grossman, 2015 | 0/0 | - | 0/1 |
|  |  | PDD < HC | 0/0 | - | 1/1 | Ash and Grossman, 2015* | 0/0 | - | 1/1 |
|  |  | LBD < HC | 0/0 | - | 1/1 | Ash and Grossman, 2015* | 0/0 | - | 1/1 |
|  |  | HD = HC | 0/1 | Jensen et al., 2006 | 0/0 | - | 0/0 | - | 0/1 |
|  | Subjects  (picture description) | HD = HC | 0/1 | Jensen et al., 2006 | 0/0 | - | 0/0 | - | 0/1 |
|  | Objects  (picture description) | HD = HC | 0/1 | Jensen et al., 2006 | 0/0 | - | 0/0 | - | 0/1 |
|  | Places  (picture description) | HD = HC | 0/1 | Jensen et al., 2006 | 0/0 | - | 0/0 | - | 0/1 |
|  | Actions  (picture description) | HD < HC | 1/1 | Jensen et al., 2006* | 0/0 | - | 0/0 | - | 1/1 |
|  | Modalizations | HD = HC | 0/1 | Jensen et al., 2006 | 0/0 | - | 0/0 | - | 0/1 |

Supplementary Table 5. Results of studies assessing linguistic variables in connected speech in Amyotrophic Lateral Sclerosis. The results are expressed as the ratio between the studies reporting significant between-group differences and the number of studies inquiring each specific feature. Dark gray rows represent variables significant in at least more than half of the studies considered for each variable (with at least three studies investigating the same feature) or in two out of two studies; the light grey rows represent the features that were tested and found significant in only one study. White rows represent variables not significant. An asterisk (*) is used to highlight the specific studies founding significant between-group differences

HC = Healthy Controls;

ALS = Amyotrophic Lateral Sclerosis.

| **LINGUISTIC LEVEL** | **FEATURE** | **RESULTS** | **PICTURE DESCRIPTION**  **Ratio References** | | **STORY** NARRATION  **Ratio References** | | **TOTAL RATIO** |
| --- | --- | --- | --- | --- | --- | --- | --- |
| **PHONETIC** | Total pause time | ALS > HC | 0/0 | - | 1/1 | Ash et al., 2014 | 1/1 |
|  | Filled pauses | ALS = HC | 0/1 | Tsermentseli et al., 2016 | 0/0 | - | 0/1 |
|  | Speech rate | ALS = HC | 0/1 | Tsermentseli et al,. 2016 | 0/2 | Ash et al., 2015; Ash et al., 2014 | 0/3 |
|  | Total Locution Time | ALS = HC | 0/1 | Tsermentseli et al,. 2016 | 0/1 | Ash et al., 2014 | 0/2 |
|  | Phonetic errors | ALS > HC | 1/1 | Tsermentseli et al,. 2016* | 0/1 | Ash et al., 2015 | 1/2 |
| **PHONOLOGICAL** | Phonemic errors | ALS = HC | 0/1 | Tsermentseli et al,. 2016 | 0/0 | - | 0/1 |
|  | False starts | ALS = HC | 0/1 | Tsermentseli et al,. 2016 | 0/0 | - | 0/1 |
| **LEXICO-SEMANTIC** | Noun rate | ALS = HC | 0/0 | - | 0/1 | Ash et al., 2015 | 0/1 |
|  | Verb rate | ALS = HC | 0/1 | Tsermentseli et al., 2016 | 0/0 | - | 0/1 |
|  | Pronoun rate | ALS = HC | 0/1 | Tsermentseli et al., 2016 | 0/0 | - | 0/1 |
|  | Closed-class words | ALS = HC | 0/1 | Tsermentseli et al., 2016 | 0/0 | - | 0/1 |
|  | Semantic errors | ALS > HC | 1/1 | Tsermentseli et al., 2016* | 0/0 | - | 1/1 |
|  | Repaired sequences | ALS = HC | 0/1 | Tsermentseli et al., 2016 | 0/1 | Ash et al.,, 2015 | 0/2 |
| **MORPHO-SYNTACTIC** | Inflected verbs | ALS = HC | 0/0 | - | 0/1 | Ash et al., 2015 | 0/1 |
| **SYNTACTIC** | **Utterances** | ALS < HC | 0/1 | Roberts-South et al., 2012 | 2/2 | Ash et al., 2014*; Ash et al., 2015* | **2/3** |
|  | **Mean length of utterance** | ALS < HC | 2/2 | Tsermentseli et al., 2016*; Roberts-South et al., 2012* | 0/0 | - | **2/2** |
|  | Incomplete sentences | ALS > HC | 1/1 | Tsermentseli et al., 2016* | 0/0 | - | 1/1 |
|  | Well-formed sentences | ALS < HC | 0/0 | - | 1/1 | Ash et al., 2015* | 1/1 |
|  | Embeddings | ALS = HC | 0/1 | Tsermentseli et al., 2016 | 0/0 | - | 0/1 |
|  | Syntactic errors | ALS = HC | 0/1 | Tsermentseli et al., 2016 | 0/0 | - | 0/1 |
| **DISCOURSE AND PRAGMATIC** | **Total words** | ALS < HC | 1/2 | Tsermentseli et al., 2016*; Roberts-South et al., 2012 | 2/2 | Ash et al., 2014*; Ash et al., 2015* | **3/4** |
|  | Local coherence | ALS < HC | 0/0 | - | 1/1 | Ash et al., 2014* | 1/1 |
|  | Global coherence | ALS = HC | 0/0 | - | 0/1 | Ash et al., 2014 | 0/1 |
|  | **Topic maintenance** | ALS < HC | 0/0 | - | 0/1 | Ash et al., 2014 | 0/1 |
|  | Information content | ALS < HC | 1/1 | Roberts-South et al., 2012* | 0/0 | - | 1/1 |
|  | **Information unit or accurate report** | ALS < HC | 1/1 | Roberts-South et al., 2012* | 1/1 | Ash et al., 2015* | **2/2** |
|  | Ratio words/information unit | ALS = HC | 0/1 | Roberts-South et al., 2012* | 0/0 | - | 0/1 |
